# Connecting the Dots between Mechanosensitive Channel Abundance, Osmotic Shock, and Survival at Single-Cell Resolution

**DOI:** 10.1128/JB.00460-18

**Published:** 2018-11-06

**Authors:** Griffin Chure, Heun Jin Lee, Akiko Rasmussen, Rob Phillips

**Affiliations:** a Department of Biology and Biological Engineering, California Institute of Technology, Pasadena, California, USA; b Department of Physics, California Institute of Technology, Pasadena, California, USA; c Institute of Medical Sciences, University of Aberdeen, Foresterhill, Aberdeen, United Kingdom; d Department of Applied Physics, California Institute of Technology, Pasadena, California, USA; Queen Mary University of London

**Keywords:** biophysics, mechanosensation, osmoregulation, quantitative methods, single cell

## Abstract

Rapid changes in extracellular osmolarity are one of many insults microbial cells face on a daily basis. To protect against such shocks, Escherichia coli and other microbes express several types of transmembrane channels that open and close in response to changes in membrane tension. In E. coli, one of the most abundant channels is the mechanosensitive channel of large conductance (MscL). While this channel has been heavily characterized through structural methods, electrophysiology, and theoretical modeling, our understanding of its physiological role in preventing cell death by alleviating high membrane tension remains tenuous. In this work, we examine the contribution of MscL alone to cell survival after osmotic shock at single-cell resolution using quantitative fluorescence microscopy. We conducted these experiments in an E. coli strain which is lacking all mechanosensitive channel genes save for MscL, whose expression was tuned across 3 orders of magnitude through modifications of the Shine-Dalgarno sequence. While theoretical models suggest that only a few MscL channels would be needed to alleviate even large changes in osmotic pressure, we find that between 500 and 700 channels per cell are needed to convey upwards of 80% survival. This number agrees with the average MscL copy number measured in wild-type E. coli cells through proteomic studies and quantitative Western blotting. Furthermore, we observed zero survival events in cells with fewer than ∼100 channels per cell. This work opens new questions concerning the contribution of other mechanosensitive channels to survival, as well as regulation of their activity.

**IMPORTANCE** Mechanosensitive (MS) channels are transmembrane protein complexes which open and close in response to changes in membrane tension as a result of osmotic shock. Despite extensive biophysical characterization, the contribution of these channels to cell survival remains largely unknown. In this work, we used quantitative video microscopy to measure the abundance of a single species of MS channel in single cells, followed by their survival after a large osmotic shock. We observed total death of the population with fewer than ∼100 channels per cell and determined that approximately 500 to 700 channels were needed for 80% survival. The number of channels we found to confer nearly full survival is consistent with the counts of the numbers of channels in wild-type cells in several earlier studies. These results prompt further studies to dissect the contribution of other channel species to survival.

## Abstract (precis)

Mechanosensitive (MS) channels are transmembrane protein complexes which open and close in response to changes in membrane tension as a result of osmotic shock. Despite extensive biophysical characterization, the contribution of these channels to cell survival remains largely unknown. In this work, we used quantitative video microscopy to measure the abundance of a single species of MS channel in single cells, followed by their survival after a large osmotic shock. We observed total death of the population with fewer than ∼100 channels per cell and determined that approximately 500 to 700 channels were needed for 80% survival. The number of channels we found to confer nearly full survival is consistent with the counts of the numbers of channels in wild-type cells in several earlier studies. These results prompt further studies to dissect the contribution of other channel species to survival.

## INTRODUCTION

**FIG 1:**
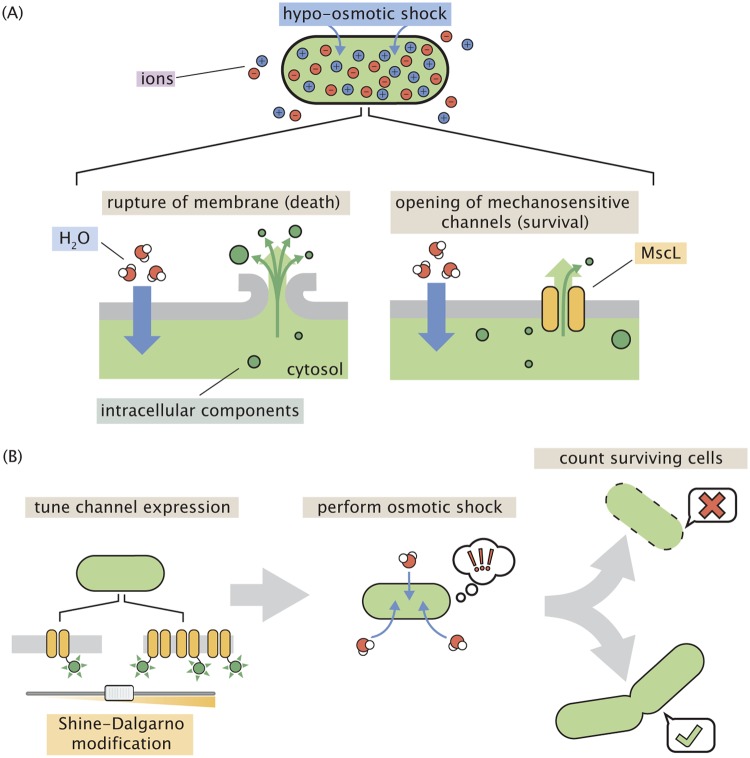
Role of mechanosensitive channels during hypo-osmotic shock. (A) A hypo-osmotic shock results in a large difference in the osmotic strength between the intracellular and extracellular spaces. As a result, water rushes into the cell to equalize this gradient, increasing the turgor pressure and tension in the cell membrane. If no mechanosensitive channels are present and membrane tension is high (left), the membrane ruptures, releasing intracellular content into the environment and resulting in cell death. If mechanosensitive channels are present (right) and membrane tension is beyond the gating tension, the mechanosensitive channel MscL opens, releasing water and small intracellular molecules into the environment, thus relieving pressure and membrane tension. (B) The experimental approach undertaken in this work. The number of mechanosensitive channels tagged with a fluorescent reporter is tuned through modification of the Shine-Dalgarno sequence of the *mscL* gene. The cells are then subjected to a hypo-osmotic shock and the number of surviving cells are counted, allowing the calculation of a survival probability.

Changes in the extracellular osmolarity can be a fatal event for the bacterial cell. Upon a hypo-osmotic shock, water rushes into the cell across the membrane, leaving the cell with no choice but to equalize the pressure. This equalization occurs either through damage to the cell membrane (resulting in death) or through the regulated flux of water molecules through transmembrane protein channels (Fig. 1A). Such proteinaceous pressure release valves have been found across all domains of life, with the first bacterial channel being described in 1987 (1). Over the past 30 years, several more channels have been discovered, described, and (in many cases) biophysically characterized. Escherichia coli, for example, has seven of these channels (one MscL and six MscS homologs), which have various degrees of conductance, gating mechanisms, and expression levels. While they have been the subject of much experimental and theoretical dissection, much remains a mystery with regard to the roles their abundance and interaction with other cellular processes play in the greater context of physiology (2–8).

Of the seven channels in E. coli, the mechanosensitive channel of large conductance (MscL) is one of the most abundant and the best characterized. This channel has a large conductance (3 nS) and mediates the flux of water molecules across the membrane via an ∼3-nm-wide pore in the open state (9, 10). Molecular dynamics simulations indicate that a single open MscL channel permits the flux of 4 × 10^9^ water molecules per second, which is an order of magnitude larger than a single aquaporin channel (BNID 100479) (11, 12). This suggests that having only a few channels per cell could be sufficient to relieve even large changes in membrane tension. Electrophysiological experiments have suggested a small number of channels per cell (13, 14); however, more recent approaches using quantitative Western blotting, fluorescence microscopy, and proteomics have measured several hundred MscL per cell (3, 15, 16). To further complicate matters, the expression profile of MscL appears to depend on the growth phase, available carbon source, and other environmental challenges (3, 16, 17). While there are likely more than just a few channels per cell, why cells seem to need so many and the biological rationale behind their condition-dependent expression both remain a mystery.

While their biochemical and biophysical characteristics have received much attention, their connection to cell survival is understudied. Drawing such a direct connection between channel copy number and survival requires quantitative *in vivo* experiments. To our knowledge, the work presented in van den Berg et al. (8) is the first attempt to simultaneously measure channel abundance and survivability for a single species of mechanosensitive channel. While the measurement of channel copy number was performed at the level of single cells using superresolution microscopy, survivability after a hypo-osmotic shock was assessed in bulk plating assays, which rely on serial dilutions of a shocked culture followed by counting the number of resulting colonies after incubation. Such bulk assays have long been the standard for querying cell viability after an osmotic challenge. While they have been highly informative, they reflect only the mean survival rate of the population, obfuscating the variability in survival of members of the population. The stochastic nature of gene expression results in a noisy distribution of MscL channels rather than a single value, meaning those cells found in the long tails of the distribution have quite different survival rates than the mean but are lost in the final calculation of survival probability.

In this work, we present an experimental system to quantitatively probe the interplay between MscL copy number and survival at single-cell resolution, as shown in Fig. 1B. We generated an E. coli strain in which all seven mechanosensitive channels had been deleted from the chromosome, followed by the chromosomal integration of a single gene encoding an MscL-superfolder green fluorescent protein (sfGFP) fusion protein. To explore copy number regimes beyond those of the wild-type expression level, we modified the Shine-Dalgarno sequence of this integrated construct, allowing us to cover nearly 3 decades of MscL copy number. To probe survivability, we exposed cells to a large hypo-osmotic shock at controlled rates in a flow cell under a microscope, allowing the observation of the single-cell channel copy number and the resulting fate of single cells. With this large set of single-cell measurements, we approach the calculation of survival probability in a manner that is free of binning bias, which allows the reasonable extrapolation of survival probability to copy numbers outside the observed range. In addition, we show that several hundred channels are needed to convey high rates of survival and observe a minimum number of channels needed to permit any degree of survival.

## RESULTS

### Quantifying the single-cell MscL copy number.

The principal goal of this work is to examine the contribution of a single mechanosensitive channel species to cell survival under a hypo-osmotic shock. While this procedure could be performed for any species of channel, we chose MscL as it is the most well characterized and one of the most abundant species in E. coli. To probe the contribution of MscL alone, we integrated an *mscL* gene encoding an MscL-superfolder GFP (sfGFP) fusion into a strain in which all seven known mechanosensitive channel genes were deleted from the chromosome (5). Chromosomal integration imposes strict control on the gene copy number compared to expression from plasmid-borne systems, which is important to minimize variation in channel expression across the population and provide conditions more representative of native cell physiology. Abrogation of activity, mislocalization, or cytotoxicity are all inherent risks associated with creating chimeric reporter constructs. In Supplement SA in the supplemental material, we carefully dissect the functionality of this protein through electrophysiology (Fig. S1), measure the rate of fluorophore maturation (Fig. S2), and quantify potential aggregates (Fig. S3 and S4). To the best of our knowledge, the MscL-sfGFP fusion protein functions identically to the wild type, allowing us to confidently draw conclusions about the physiological role this channel plays in wild-type cells.

**FIG 2:**
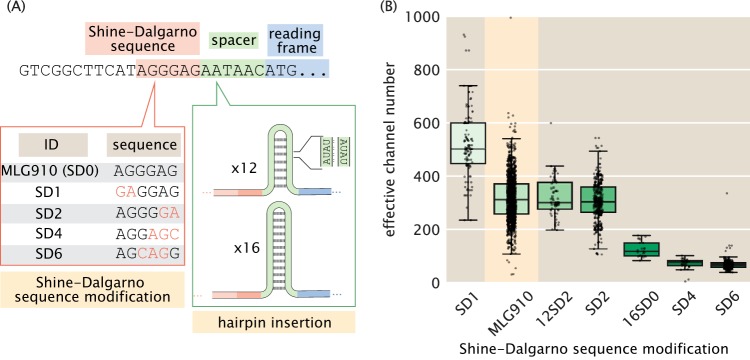
Control of MscL expression and calculation of channel copy number. (A) Schematic view of the expression modifications performed in this work. The beginning portion of the native *mscL* sequence is shown with the Shine-Dalgarno sequence, spacer region, and start codon shaded in red, green, and blue, respectively. The Shine-Dalgarno sequence was modified through the Salis laboratory's RBS (Ribosome Binding Site) Calculator (18, 19). The wild-type sequence (MLG910) is shown in black, with mutations for the four Shine-Dalgarno mutants highlighted in red. Expression was further modified by the insertion of repetitive AT bases into the spacer region, generating hairpins of various length which acted as a thermodynamic barrier for translation initiation. (B) Variability in effective channel copy number is computed using the standard candle. The boxes represent the interquartile region of the distribution, the center line displays the median, and the whiskers represent 1.5 times the maximum and minimum of the interquartile region. Individual measurements are denoted as black points. Data for the strain used for calibration of channel copy number (MLG910) are highlighted in yellow.

To modulate the number of MscL channels per cell, we developed a series of mutants which were designed to decrease the expression relative to that of the wild type. These changes involved direct alterations of the Shine-Dalgarno sequence, as well as the inclusion of AT hairpins of various lengths directly upstream from the start codon, which influences the translation rate and, hence, the number of MscL proteins produced (Fig. 2A). The six Shine-Dalgarno sequences used in this work were chosen using the RBS (ribosome binding site) Calculator from the Salis laboratory at the Pennsylvania State University (18, 19). While the designed Shine-Dalgarno sequence mutations decreased the expression relative to that of the wild type as intended, the distribution of expression levels was remarkably wide, spanning an order of magnitude.

To measure the number of MscL channels per cell, we determined a fluorescence calibration factor to translate arbitrary fluorescence units per cell to protein copy numbers. While there have been numerous techniques developed over the past decade to directly measure this calibration factor, such as quantifying single-molecule photobleaching constants or measuring the binomial partitioning of fluorescent proteins upon cell division (3, 20), we used a priori knowledge of the mean MscL-sfGFP expression level of a particular E. coli strain to estimate the average fluorescence of a single channel. In the work of Bialecka-Fornal et al. (3), the authors used single-molecule photobleaching and quantitative Western blotting to probe the expression of MscL-sfGFP under a wide range of growth conditions. To compute a calibration factor, we used strain MLG910 [E. coli K-12 MG1655 ϕ(*mscL*-sfGFP)] as a “standard candle,” highlighted in yellow in Fig. 2B. This standard-candle strain was grown and imaged under conditions identical to those under which the MscL count was determined through fluorescence microscopy. The calibration factor was computed by dividing the mean total cell fluorescence by the known MscL copy number, resulting in a measure of arbitrary fluorescence units per MscL channel. Details regarding this calculation and appropriate propagation of error, as well as its sensitivity to various growth media, can be found in Materials and Methods, as well as in Supplement SB (Fig. S5 to S8).

While it is seemingly straightforward to use this calibration factor to determine the total number of channels per cell for wild-type or highly expressing strains, the calculation for the lowest-expressing strains is complicated by distorted cell morphology. We observed that, as the channel copy number decreased, cellular morphology became increasingly aberrant, with filamentous, bulging, and branched cells becoming more abundant (Fig. S7A). This morphological defect has been observed when altering the abundance of several species of mechanosensitive channels, suggesting that they play an important role in general architectural stability (3, 4). As these aberrant morphologies can vary widely in size and shape, calculating the number of channels per cell becomes a more nuanced endeavor. For example, taking the total MscL copy number for these cells could skew the final calculation of survival probability, as a large but severely distorted cell would be interpreted as having more channels than a smaller, wild-type-shaped cell (Fig. S7B). To correct for this pathology, we computed the average expression level per unit area for each cell and multiplied this by the average cellular area of our standard-candle strain, which is morphologically indistinguishable from wild-type E. coli, allowing the calculation of an effective channel copy number. The effect of this correction can be seen in Fig. S7C and D, which illustrate that there is no other correlation between cell area and channel expression.

Our calculation of the effective channel copy number for our suite of Shine-Dalgarno mutants is shown in Fig. 2B. The expression levels of these strains cover nearly 3 orders of magnitude, with the extremes ranging from approximately 4 channels per cell to nearly 1,000. While the mean values of each strain are somewhat distinct, the distributions show a large degree of overlap, making one strain nearly indistinguishable from another. This variance is a quantity that is lost in the context of bulk scale experiments but can be accounted for via single-cell methods.

### Performing a single-cell hypo-osmotic challenge assay.

**FIG 3:**
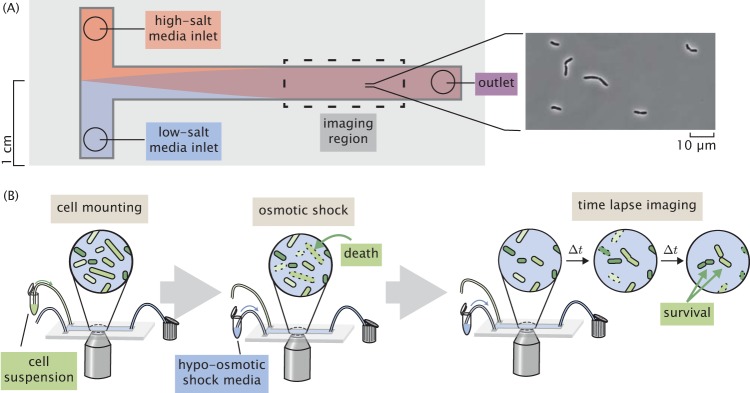
Experimental approach to measuring survival probability. (A) Layout of a home-made flow cell for subjecting cells to osmotic shock. Cells are attached to a polyethylenimine-functionalized surface of a glass coverslip within the flow chamber by loading a dilute cell suspension through one of the inlets. (B) The typical experimental procedure. Cells are loaded into a flow chamber as shown in panel A and mounted to the glass coverslip surface. Cells are subjected to a hypo-osmotic shock by flowing hypotonic medium into the flow cell. After shock, the cells are monitored for several hours and surviving cells are identified.

To measure the channel copy number of a single cell and query its survival after a hypo-osmotic shock, we used a custom-made flow cell in which osmotic shock and growth can be monitored in real time using video microscopy (Fig. 3A). The design and characterization of this device have been described in depth previously (4) and are briefly described in Materials and Methods. Using this device, cells were exposed to a large hypo-osmotic shock by switching between LB Lennox medium supplemented with 500 mM NaCl and LB Lennox medium alone. Cells containing the six Shine-Dalgarno modifications shown in Fig. 2B (excluding MLG910) were subjected to hypo-osmotic shocks at controlled rates while under observation. After the application of the osmotic shock, the cells were imaged every 60 s for 4 to 6 h. Each cell was monitored over the outgrowth period and was manually scored as a survivor, a fatality, or inconclusive. The criteria used for scoring death were the same as those previously described by Bialecka-Fornal et al. (4). Survivors were defined as cells that underwent multiple divisions postshock. To qualify as survivors, cells must undergo at least two divisions, although more typically, four to eight divisions are observed without any signs of slowing down. Imaging was stopped when the survivor cells began to go out of focus or overlap each other. Survivors do not show any sign of ceasing division. More information regarding this classification can be found in Materials and Methods, as well as in Supplement SC (Fig. S9 to S10 and Tables S1 and S2). The experimental protocol can be seen in brief in Fig. 3B.

**FIG 4:**
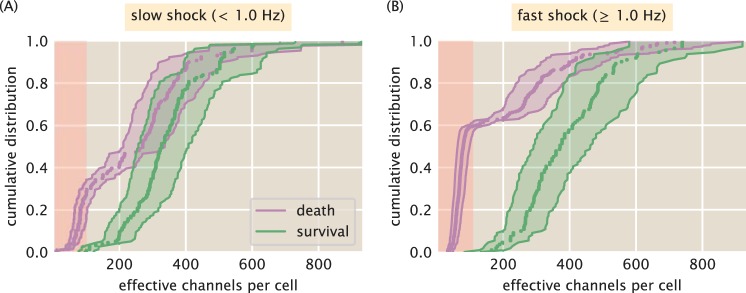
Distributions of survival and death as functions of effective channel number. (A) Empirical cumulative distributions of channel copy numbers separated by survival or death after a slow (<1.0 Hz) osmotic shock. (B) Empirical cumulative distributions for a fast (≥1.0 Hz) osmotic shock. Shaded green and purple regions represent the 95% credible region of the effective channel number calculation for each cell. The shaded red stripe signifies the range of channels in which no survival was observed.

Due to the extensive overlap in expression levels between the different Shine-Dalgarno mutants (Fig. 2B), computing the survival probability by treating each mutant as an individual bin obfuscates the relationship between channel abundance and survival. To more thoroughly examine this relationship, all measurements were pooled, with each cell being treated as an individual experiment. The hypo-osmotic shock applied in these experiments was varied across a range of 0.02 Hz (complete exchange in 50 s) to 2.2 Hz (complete exchange in 0.45 s). Rather than pooling this wide range of shock rates into a single data set, we chose to separate the data into “slow shock” (<1.0 Hz) and “fast shock” (≥1.0 Hz) classes. Other groupings of shock rates were explored and are discussed in Supplement SD (Fig. S11 and S12). The cumulative distributions of channel copy numbers separated by survival rates are shown in Fig. 4. In these experiments, survival was never observed for a cell containing fewer than approximately 100 channels per cell, indicated by the red stripes in Fig. 4. This suggests that there is a minimum number of channels needed for survival that is on the order of 100 per cell. We also observe a slight shift in the surviving fraction of the cells toward higher effective copy numbers, which matches our intuition that including more mechanosensitive channels increases the survival probability.

### Prediction of survival probability as a function of channel copy number.

There are several ways by which the survival probability can be calculated. The most obvious approach would be to group each individual Shine-Dalgarno mutant as a single bin and compute the average MscL copy number and the survival probability. Binning by strain is the most frequently used approach for such measurements and has provided valuable insight into the qualitative relationship of survival to other physiological factors (4, 8). However, the copy number distribution for each Shine-Dalgarno mutant (Fig. 2B) is remarkably wide and overlaps with those of the other strains. We argue that this coarse-grained binning negates the benefits of performing single-cell measurements, as two strains with different means but overlapping quartiles would be treated as distinctly different distributions.

Another approach would be to pool all data, irrespective of the Shine-Dalgarno mutation, and bin by a defined range of channels. Depending on the width of the bin, this could allow for finer resolution of the quantitative trend, but the choice of the bin width is arbitrary with the a priori knowledge that is available. Drawing a narrow bin width can easily restrict the number of observed events to small numbers where the statistical precision of the survival probability is lost. On the other hand, drawing wide bins increases the precision of the estimate but becomes further removed from a true single-cell measurement and represents a population mean, even though it may be a smaller population than binning by the Shine-Dalgarno sequence alone. In both of these approaches, it is difficult to extrapolate the quantitative trend outside the experimentally observed region of channel copy number. Here, we present a method to estimate the probability of survival for any channel copy number, even those that lie outside the experimentally queried range.

To quantify the survival probability while maintaining single-cell resolution, we chose to use a logistic regression model which does not require grouping data into arbitrary bins and treats each cell measurement as an independent experiment. Logistic regression is an inferential method to model the probability of a Boolean or categorical event (such as survival or death) given one or several predictor variables and is commonly used in medical statistics to compute survival rates and dose-response curves (21, 22). The primary assumption of logistic regression is that the log-odds probability of survival *p_s_* is linearly dependent on the predictor variable, in our case the log channels per cell *N_c_* with a dimensionless intercept β_0_ and slope β_1_,

(1) $$\log\;\frac{p_{s}}{1–p_{s}}=\beta_{0}+\beta_{1}\log\;N_{c}$$

Under this assumption of linearity, β_0_ is the log-odds probability of survival with no MscL channels. The slope β_1_ represents the change in the log-odds probability of survival conveyed by a single channel. As the calculated number of channels in this work spans nearly 3 orders of magnitude, it is better to perform this regression on log *N_c_*, as regressing on *N_c_* directly would give undue weight to lower channel copy numbers due to the sparse sampling of high-copy-number cells. The functional form shown in equation 1 can be derived directly from Bayes' theorem and is shown in Supplement SE. If one knows the values of β_0_ and β_1_, the survival probability can be expressed as

(2) $$p_{s}=\frac{1}{1+N_{c}^{-\beta_{1}}e^{-\beta_{0}}}$$

In this analysis, we used Bayesian inferential methods to determine the most likely values of the coefficients (described in detail in Fig. S13 and S14 in Supplement SE).

**FIG 5:**
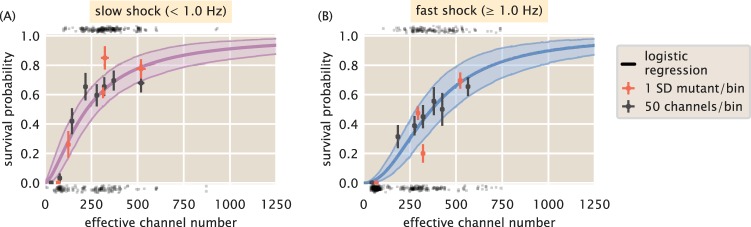
Probability of survival as a function of MscL copy number. (A) Estimated survival probability for survival under slow shock as a function of channel copy number. (B) Estimated survival probability of survival under a fast shock as a function of channel copy number. Solid curves correspond to the most probable survival probability from a 1-dimensional logistic regression. Shaded regions represent the 95% credible regions. Points at the top and bottom of plots represent measurements of individual cells which survived and perished, respectively. The red and black points within the plots correspond to the survival probabilities estimated via binning by Shine-Dalgarno sequence and binning by groups of 50 channels per cell, respectively. Horizontal error bars represent the standard errors of the means from at least 25 measurements. Vertical error bars represent the certainty of the probability estimate given *n* survival events from *N* total observations.

The results of the logistic regression are shown in Fig. 5. We see a slight rightward shift of the survival probability curve under fast shock relative to the case for slow shock, reaffirming the conclusion that survival is also dependent on the rate of osmotic shock (4). This rate dependence has been observed for cells expressing MscL alongside other species of mechanosensitive channels but not for MscL alone. This suggests that MscL responds differently to different rates of shock, highlighting the need for further study of rate dependence and the coordination between different species of mechanosensitive channels. The results in Fig. 5 also show that several hundred channels are required to provide appreciable protection from osmotic shock. For a survival probability of 80%, a cell must have approximately 500 to 700 channels per cell for a fast and a slow shock, respectively. The results from the logistic regression are shown as continuous colored curves in Fig. 5; the individual cell measurements separated by survival and death are shown at the top and bottom of each plot, respectively, and are included to provide a sense of sampling density.

Over the explored range of MscL copy numbers, we observed a maximum of 80% survival for any binning method. The remaining 20% survival may be attained when the other species of mechanosensitive channels are expressed alongside MscL. However, it is possible that the flow cell method performed in this work lowers the maximal survival fraction, as the cells are exposed to several, albeit minor, mechanical stresses, such as loading into the flow cell and chemical adherence to the glass surface. To ensure that the results from logistic regression accurately describe the data, we can compare the survival probabilities to those obtained using the binning methods described earlier (Fig. 5, red and black points). Nearly all binned data fall within the error of the prediction (see Materials and Methods for definition of error bars in probability data), suggesting that this approach accurately reflects the survival probability and giving license to extrapolate the estimation of survival probability to regions outside our experimentally explored copy number regime.

Thus far, we have dictated that for a given rate of osmotic shock (i.e., “fast” or “slow”), the survival probability is dependent only on the number of channels. In Fig. S13, we show the results of including other predictor variables, such as area and shock rate alone. In such cases, including other predictors resulted in pathological curves, showing that channel copy number is the most informative of the available predictor variables.

## DISCUSSION

One of the most challenging endeavors in the biological sciences is linking the microscopic details of cellular components to the macroscale physiology of the organism. This formidable task has been undertaken repeatedly in the recent history of biology, especially in the era of DNA sequencing and single-molecule biochemistry. For example, the scientific community has been able to connect sickle-cell anemia to a single amino acid substitution in hemoglobin which promotes precipitation under a change in O_2_ partial pressure (23–25). Others have assembled a physical model that quantitatively describes chemosensation in bacteria (26), in which the arbiter of sensory adaptation is the repeated methylation of chemoreceptors (27–30). In the past ∼50 years alone, numerous biological and physical models of the many facets of the central dogma have been assembled that give us a sense of the interplay between the genome and physiology. For example, the combination of biochemical experimentation and biophysical models have given us a picture of how gene dosage affects furrow positioning in Drosophila (31), how recombination of V(D)J gene segments generates an extraordinarily diverse antibody repertoire (32–34), and how telomere shortening through DNA replication is intrinsically tied to cell senescence (35, 36), to name just a few of many such examples.

By no means are we finished with any of these topics. Rather, it is quite the opposite, in the sense that having a handle on the biophysical knobs that tune the behavior opens the door to a litany of new scientific questions. In the case of mechanosensation and osmoregulation, we have only recently been able to determine some of the basic facts that allow us to approach this fascinating biological phenomenon biophysically. The dependence of survival on mechanosensitive channel abundance is a key quantity that is missing from our collection of critical facts. To our knowledge, this work represents the first attempt to quantitatively control the abundance of a single species of mechanosensitive channel and examine the physiological consequences in terms of survival probability at single-cell resolution. Our results reveal two notable quantities. First, out of the several hundred single-cell measurements, we never observed a cell which had fewer than approximately 100 channels per cell and survived an osmotic shock, irrespective of the shock rate. The second is that between 500 and 700 channels per cell are needed to provide ≥80% survival, depending on the shock rate.

Only recently has the relationship between the MscL copy number and the probability of survival been approached experimentally. In the work of van den Berg et al., the authors examined the contribution of MscL to survival in a genetic background where all other known mechanosensitive channels had been deleted from the chromosome and plasmid-borne expression of an MscL-mEos3.2 fusion was tuned through an isopropyl-β-d-thiogalactopyranoside (IPTG)-inducible promoter (8). In that work, they measured the single-cell channel abundance through superresolution microscopy and queried survival through bulk assays. They report a nearly linear relationship between survival and copy number, with approximately 100 channels per cell conveying 100% survival. This number is significantly smaller than our observation of approximately 100 channels as the minimum number needed to convey any observable degree of survival.

The disagreement between the numbers reported in this work and by van den Berg et al. (8) may arise partially from subtle differences in the experimental approach. The primary practical difference is the magnitude of the osmotic shock. van den Berg et al. (8) applied an approximately 600-mosmol downshock in bulk, whereas we applied a 1-osmol downshock, which would lead to lower survival (37). In their work, the uncertainty in both the MscL channel count and survival probability is roughly 30% (Fig. S14 in the supplemental material). Given this uncertainty, it is reasonable to interpret that the number of channels needed for complete protection from osmotic downshock is between 100 and 250 per cell. The uncertainty in determining the number of channels per cell is consistent with the observed width of the channel number distribution of the Shine-Dalgarno sequence mutants used in this work (Fig. 2B). A unique property of the single-cell measurements performed in this work is the direct observation of the survival or death of individual cells. We find that the results of morphological classification and classification through propidium iodide staining agree within 1% (see Supplement SC in the supplemental material). Bulk plating assays, such as are used by van den Berg et al. (8), rely on colony formation and outgrowth to determine survival probability. As is reported in their supplemental information, the precision in this measurement is around 30% (Fig. S14). Accounting for this uncertainty brings both measurements within a few fold, where we still consistently observe lower survival for a given channel number. This remaining disagreement may be accounted for by systematic uncertainty in both experimental methods.

For example, variations in the lengths of outgrowth, shock rates, and counting statistics could lead to a bias toward higher observed survival rates in ensemble plating assays. During the outgrowth phase, the control sample not exposed to an osmotic shock is allowed to grow for approximately 30 min in a high-salt medium before plating. The shocked cells, however, are allowed to grow in a low-salt medium. We have found that the difference between the growth rates under these two conditions can be appreciable (approximately 35 min versus 20 min, respectively), as can be seen by the results in Fig. S2. Cells that survived an osmotic shock may have a growth advantage relative to the control sample if the shock-induced lag phase is less than the outgrowth, leading to higher observed survival rates (37). This is one possible explanation for the survival rates in excess of 100% that are reported. Cells that survived an osmotic shock may have a growth advantage relative to the normalization sample if the shock-induced lag phase is less than the outgrowth, leading to higher observed survival rates, even surpassing 100%. We have performed these assays ourselves and have observed survival rates above 100% (ranging from 110% to 125%) with an approximately 30% error (see Fig. S3 in the work of Bialecka-Fornal et al. [3]), which we concluded arose from differences in growth rate. We also note that survival rates greater than 100% are observed by van den Berg et al. (Fig. S14) (8). For strains that have survival rates between 80% and 100%, the uncertainty is typically large, making it difficult to make precise statements regarding when full survival is achieved.

It has been shown that there is a strong inverse relationship between the rate of osmotic shock and survival probability (4). Any experiment in which the shock was applied more slowly or quickly than in another would bias toward higher or lower survivability, respectively. The shocks applied in bulk assays are often performed manually, which can be highly variable. We note that in our experiments, we frequently observed cells which did not separate and formed chains of two or more cells (Fig. S9 and S10). In plating assays, it is assumed that colonies arise from a single founding cell; however, a colony formed by a cluster of living and dead cells would be interpreted as a single surviving cell, effectively masking the death of the others in the CFU. This too could bias the measurement toward higher survival rates. Single-cell shock experiments can also have systematic errors which can bias the results toward lower survival rates. Such errors are associated with handling of the cells, such as loading into the flow cell, which may cause shear damage, adhering the cells to the coverslip, and any chemical perturbations introduced by the dye used to measure the shock rate.

Despite these experimental differences, the results of this work and of van den Berg et al. (8) are in agreement that MscL must be present at the level of 100 or more channels per cell in wild-type cells to convey appreciable survival. As both of these works were performed in a strain in which the only mechanosensitive channel was MscL, it remains unknown how the presence of the other channel species would alter the number of MscL needed for complete survival. In our experiments, we observed a maximum survival probability of approximately 80% even with close to 1,000 MscL channels per cell. It is possible that the combined efforts of the six other mechanosensitive channels would make up for some if not all of the remaining 20%. To explore the contribution of another channel to survival, van den Berg et al. (8) also queried the contribution of MscS, another mechanosensitive channel, to survival in the absence of any other species of mechanosensitive channel. It was found that over the explored range of MscS channel copy numbers, the maximum survival rate was approximately 50%, suggesting that different mechanosensitive channels have an upper limit to how much protection they can confer. Both the results of van den Berg et al. (8) and our work show that there is still much to be learned with respect to the interplay between the various species of mechanosensitive channel, as well as their regulation.

Recent work has shown that both the magnitude and the rate of osmotic downshock are important factors in determining cell survival (4). In this work, we show that this finding holds true for a single species of mechanosensitive channel, even at high levels of expression. One might naively expect that this rate-dependent effect would disappear once a certain threshold of channels had been met. Our experiments, however, show that even at nearly 1,000 channels per cell, the predicted survival curves for a slow (<1.0 Hz) and fast (≥1.0 Hz) shock are shifted relative to each other, with the fast shock predicting lower rates of survival. This suggests that either we have not reached this threshold in our experiments or there is more to understand about the relationship between the abundance, channel species, and shock rate.

Some experimental and theoretical treatments suggest that only a few copies of MscL or MscS should be necessary for 100% protection, given our knowledge of the conductance and the maximal water flux through the channel in its open state (11, 38). However, recent proteomic studies have revealed average MscL copy numbers to be in the range of several hundred per cell, depending on the condition, as can be seen by the data in Table 1 (15, 16, 39). Studies focusing solely on MscL have shown similar counts through quantitative Western blotting and fluorescence microscopy (3). Electrophysiology studies have told another story, with copy number estimates ranging between 4 and 100 channels per cell (17, 40). These measurements, however, measure the number of active channels. The factors regulating channel activity in these experiments could include perturbations during the sample preparation or reflect some unknown mechanism of regulation, such as the presence or absence of interacting cofactors (41). The work described here, on the other hand, measures the maximum number of channels that could be active and may be able to explain why the channel abundance is higher than estimated by theoretical means. There remains much more to be learned about the regulation of activity in these systems. As the *in vivo* measurement of protein copy number becomes accessible through novel single-cell and single-molecule methods, we will continue to collect more facts about this fascinating system and hopefully connect the molecular details of mechanosensation with perhaps the most important physiological response—life or death.

**TABLE 1:** Measured cellular copy numbers of MscL

Reported no. of channels per cell	Method	Reference
480 ± 103^*a*^	Western blotting	3
560^*b*^	Ribosomal profiling	39
331^*b*^	Mass spectrometry	15
583^*b*^	Mass spectrometry	16
4 or 5	Electrophysiology	17
10–100	Electrophysiology	13
10–15	Electrophysiology	40

a Mean value ± standard deviation.

b MscL channel copy number was inferred from the total number of MscL peptides detected.

## MATERIALS AND METHODS

### Bacterial strains and growth conditions.

The bacterial strains are described in Table S3 in the supplemental material. The parent strain for the mutants used in this study was MJF641 (5) (generously provided by Samantha Miller and Ian Booth), a strain which had all seven mechanosensitive channels deleted. The MscL-sfGFP-coding region from MLG910 (3) was integrated into MJF641 by P1 transduction, creating strain D6LG-Tn10. Selection pressure for MscL integration was created by incorporating an osmotic shock into the transduction protocol, which favored the survival of MscL-expressing strains relative to MJF641 by ∼100-fold. Screening for integration candidates was based on the fluorescence expression of plated colonies. Successful integration was verified by sequencing. Attempts to transduce RBS-modified MscL-sfGFP-coding regions became increasingly inefficient as the targeted expression level of MscL was reduced. This was due to the decreasing fluorescence levels and survival rates of the integration candidates. Consequently, Shine-Dalgarno sequence modifications were made by inserting DNA oligonucleotides with lambda red-mediated homologous recombination, i.e., recombineering (42). The oligonucleotides had a designed mutation (Fig. 2) flanked by ∼25 bp that matched the targeted MscL region (Table S4). A two-step recombineering process of selection followed by counterselection using a *tetA-sacB* gene fusion cassette (43) was chosen because of its capabilities to integrate with efficiencies comparable to those of P1 transduction and not leave antibiotic resistance markers or scar sequences in the final strain. To prepare strain D6LG-Tn10 for this scheme, the Tn*10* transposon containing the *tetA* gene needed to be removed to avoid interference with the *tetA-sacB* cassette. Tn*10* was removed from the middle of the *ycjM* gene with the primer Tn10delR (Table S2) by recombineering, creating strain D6LG (SD0). Counterselection against the *tetA* gene was promoted by using agar medium with fusaric acid (43, 44). The *tetA-sacB* cassette was PCR amplified out of strain XTL298 using primers MscLSPSac and MscLSPSacR (Table S2). The cassette was integrated in place of the spacer region in front of the MscL start codon of D6LG (SD0) by recombineering, creating the intermediate strain D6LTetSac. Positive selection for cassette integration was provided by agar medium with tetracycline. Finally, the RBS-modifying oligonucleotides were integrated by replacing the *tetA-sacB* cassette by recombineering. Counterselection against both *tetA* and *sacB* was ensured by using agar medium with fusaric acid and sucrose (43), creating the Shine-Dalgarno mutant strains used in this work.

Strain cultures were grown in 5 ml of LB Lennox medium with antibiotic (apramycin) overnight at 37°C. The next day, 50 μl of overnight culture was inoculated into 5 ml of LB Lennox medium with antibiotic and the culture was grown to an optical density at 600 nm (OD_600_) of ∼0.25. Subsequently, 500 μl of that culture was inoculated into 5 ml of LB Lennox medium supplemented with 500 mM NaCl and the culture was regrown to an OD_600_ of ∼0.25. A 1-ml aliquot was taken and used to load the flow cell.

### Flow cell.

All experiments were conducted in a home-made flow cell as shown in Fig. 3A. This flow cell has two inlets that allow media of different osmolarities to be exchanged over the course of the experiment. The imaging region is approximately 10 mm wide and 100 μm in depth. All imaging took place within 1 to 2 cm of the outlet to avoid imaging cells within a nonuniform gradient of osmolarity. The interior of the flow cell was functionalized with a 1:400 dilution of polyethylenimine prior to the addition of cells, with the excess washed away with water. A dilute cell suspension in LB Lennox medium with 500 mM NaCl was loaded into one inlet, while the other was connected to a vial of LB medium with no NaCl. This hypotonic medium was clamped during the loading of the cells.

Once the cells had adhered to the polyethylenimine-coated surface, the excess cells were washed away with the 500 mM NaCl growth medium, which was followed by a small (∼20 μl) air bubble. This air bubble forced the cells to lie flat against the imaging surface, improving the time-lapse imaging. Over the observation period, cells not exposed to an osmotic shock were able to grow for 4 to 6 divisions, showing that the flow cell does not directly impede cell growth.

### Imaging conditions.

All imaging was performed in a flow cell held at 30°C on a Nikon Ti-Eclipse microscope outfitted with a perfect focus system enclosed in a Haison environmental chamber (approximately 1°C regulation efficiency). The microscope was equipped with a 488-nm laser excitation source (CrystaLaser) and a 520/35 laser optimized filter set (Semrock). The images were collected on an Andor iXon EM+ 897 electron-multiplying charge-coupled device (EMCCD) camera, and all microscope and acquisition operations were controlled via the open-source μManager microscope control software (45). Once cells were securely mounted onto the surface of the glass coverslip, between 15 and 20 positions containing 5 to 10 cells were marked and the coordinates recorded. At each position, a phase-contrast and a GFP fluorescence image were acquired for segmentation and subsequent measurement of channel copy number. To perform the osmotic shock, LB medium containing no NaCl was pulled into the flow cell through a syringe pump. To monitor the medium exchange, both the high-salt and no-salt LB media were supplemented with a low-affinity version of the calcium-sensitive dye Rhod-2 (250 nM; TEF Labs), which fluoresces when bound to Ca^2+^. The no-salt medium was also supplemented with 1 μM CaCl_2_ to make the medium mildly fluorescent, and the exchange rate was calculated by measuring the fluorescence increase across an illuminated section of one of the positions. These images were collected in real time for the duration of the shock. The difference in measured fluorescence between the preshock images and those at the end of the shock set the scale of a 500 mM NaCl downshock. The rate was calculated by fitting a line to the middle region of this trace. Further details regarding this procedure can be found in the work of Bialecka-Fornal et al. (4).

### Image processing.

Images were processed using a combination of automated and manual methods. First, the expression of MscL was measured via segmenting individual cells or small clusters of cells in phase contrast and computing the mean pixel value of the fluorescence image for each segmented object. The fluorescence images were passed through several filtering operations which reduced high-frequency noise and corrected for uneven illumination of the excitation wavelength.

Survival or death classification was performed manually using the CellProfiler plug-in for ImageJ software (NIH). A survivor was defined as a cell which was able to undergo at least two division events after the osmotic downshock. Cell death was recognized by stark changes in cell morphology, including loss of phase contrast through ejection of cytoplasmic material, structural decomposition of the cell wall and membrane, and the inability to divide. To confirm that these morphological cues corresponded with cell death, we probed cell viability on a subset of our strains after osmotic shock through staining with propidium iodide, a DNA intercalating dye commonly used to identify dead cells (LIVE/DEAD BacLight bacterial-cell viability assay; Thermo Fisher). We found that our classification based on morphology agreed with that based on staining within 1%. More information regarding these experiments can be found in Supplement SC. Cells which detached from the surface during the postshock growth phase or those which became indistinguishable from other cells due to clustering were not counted as surviving or dead and were removed from the data set completely. A region of the cell was manually marked with 1.0 (survival) or 0.0 (death) by clicking on the image. The *xy* coordinates of the click and the assigned value were saved as an .xml file for that position.

The connection between the segmented cells and their corresponding manual markers was automated. As the manual markings were made on the first phase-contrast image after the osmotic shock, small shifts in the positions of the cell made one-to-one mapping with the segmentation mask nontrivial. The linkages between segmented cell and manual marker were made by computing all pairwise distances between the manual marker and the segmented cell centroid, taking the shortest distance as the true pairing. The linkages were then inspected manually and incorrect mappings were corrected as necessary.

All relevant statistics about the segmented objects, as well as the sample identity, date of acquisition, osmotic shock rate, and camera exposure time, were saved as .csv files for each individual experiment. A more in-depth description of the segmentation procedure and the relevant code can be accessed as a Jupyter Notebook at (http://rpgroup.caltech.edu/mscl_survival).

### Calculation of effective channel copy number.

To compute the MscL channel copy number, we relied on measuring the fluorescence level of a bacterial strain in which the mean MscL channel copy number was known via fluorescence microscopy (3). E. coli strain MLG910, which expresses the MscL-sfGFP fusion protein from the wild-type Shine-Dalgarno sequence, was grown under conditions identical to those described by Bialecka-Fornal et al. (4) in LB Miller medium (BD Medical Sciences) to an OD_600_ of ∼0.3. The cells were then diluted 10-fold, immobilized on a rigid 2% agarose substrate, placed on a glass-bottom petri dish, and imaged under the same conditions as described previously.

Images were taken of six biological replicates of MLG910 and were processed identically to those in the osmotic shock experiments. A calibration factor between the average cell fluorescence level and mean MscL copy number was then computed. We assumed that all measured fluorescence (〈*I*_tot_〉) (after filtering and background subtraction) was derived from the MscL-sfGFP fusion,

(3) $$〈I_{\text{tot}}〉=\alpha 〈N〉$$

in which α is the calibration factor and 〈*N*〉 is the mean cellular MscL-sfGFP copy number as reported by Bialecka-Fornal et al. (3). To correct for errors in segmentation, the intensity was computed as an areal density (〈*I_A_*〉) and was multiplied by the average cell area (〈*A*〉) of the population. The calibration factor was therefore computed as

(4) $$\alpha =\frac{〈I_{A}〉〈A〉}{〈N〉}$$

We used Bayesian inferential methods to compute this calibration factor, taking measurement error and replicate-to-replicate variation into account. The resulting average cell area and calibration factor were used to convert the measured cell intensities from the osmotic shock experiments to cell copy number. The details of this inference are described in depth in Supplement SB in the supplemental material.

### Logistic regression.

We used Bayesian inferential methods to find the most probable values of the coefficients β_0_ and β_1_ and the appropriate credible regions (described in detail in Supplement SD in the supplemental material). Briefly, we used Markov chain Monte Carlo (MCMC) to sample from the log posterior distribution and took the mean value of the samples for each parameter as the most probable value. MCMC was performed using the Stan probabilistic programming language (46), and all models can be found in the GitHub repository (http://github.com/rpgroup-pboc/mscl_survival).

### Calculation of survival probability error.

The vertical error bars for the points shown in Fig. 5 represent our uncertainty in the survival probability given our measurement of *n* survivors out of a total *N* single-cell measurements. The probability distribution of the survival probability *p_s_* given these measurements can be written using Bayes' theorem as

(5) $$g\left(p_{s}|n,N\right)=\frac{f\left(n|p_{s},N\right)g\left(p_{s}\right)}{f\left(n|N\right)}$$

where *g* and *f* represent probability density functions over parameters and data, respectively. The likelihood *f(n* ∣ *p_s_*, *N*) represents the probability of measuring *n* survival events, given a total of *N* measurements each with a probability of survival *p_s_*. This matches the story for the binomial distribution and can be written as

(6) $$f\left(n|p_{s},N\right)=\frac{N!}{n!\left(N–n\right)!}p_{s}^{n}{\left(1–p_{s}\right)}^{N–n}$$

To maintain maximal ignorance, we can assume that any value for *p_s_* is valid that is in the range [0, 1]. This prior knowledge, represented by *g(p_s_*), can be written as

(7) $$g\left(p_{s}\right)=\{\begin{array}{cc} 1 & 0\leq p_{s}\leq 1\\ 0 & \text{otherwise} \end{array}$$

We can also assume maximal ignorance for the total number of survival events we could measure given *N* observations, *f(n* ∣ *N*). Assuming all observations are equally likely, this can be written as

(8) $$f\left(n|N\right)=\frac{1}{N+1}$$

where the addition of one comes from the possibility of observing zero survival events. Combining equations 6, 7, and 8, the posterior distribution *g(p_s_* ∣ *n*, *N*) is

(9) $$g\left(p_{s}|n,N\right)=\frac{\left(N+1\right)!}{n!\left(N–n\right)!}p_{s}^{n}{\left(1–p_{s}\right)}^{N–n}$$

The most probable value of *p_s_*, where the posterior probability distribution given by equation 9 is maximized, can be found by computing the point at which the derivative of the log posterior with respect to *p_s_* goes to zero,

(10) $$\frac{d\log\;g\left(p_{s}|n,N\right)}{dp_{s}}=\frac{n}{p_{s}}–\frac{N–n}{1–p_{s}}=0$$

Solving equation 10 for *p_s_* gives the most likely value for the probability,

(11) $$p_{s}^{*}=\frac{n}{N}$$

So long as $N\;\gg \;{np}_{s}^{*}$, equation 9 can be approximated as a Gaussian distribution with a mean $p_{s}^{*}$ and a variance $\sigma_{p_{s}}^{2}$. By definition, the variance of a Gaussian distribution is computed as the negative reciprocal of the second derivative of the log posterior evaluated at $ps=p_{s}^{*}$,

(12) $$\sigma_{p_{s}}^{2}=-{\left(\frac{d^{2}\log\;g\left(p_{s}|n,N\right)}{dp_{s}^{2}}{|}_{p_{s}\;=\;p_{s}^{*}}\right)}^{-1}$$

Evaluating equation 12 yields

(13) $$\sigma_{p_{s}}^{2}=\frac{n\left(N–n\right)}{N^{3}}$$

Given equations 11 and 13, the most likely survival probability and estimate of the uncertainty can be expressed as

(14) $$p_{s}=p_{s}^{*}\pm \sigma_{p_{s}}$$

### Data and software availability.

All raw image data are freely available and are stored on the CaltechDATA Research Data Repository (47). The raw Markov chain Monte Carlo samples are stored as .csv files on CaltechDATA (48). All processed experimental data and Python and Stan code used in this work are freely available through our GitHub repository (http://github.com/rpgroup-pboc/mscl_survival) (49), accessible through https://doi.org/10.5281/zenodo.1252524. The scientific community is invited to fork our repository and open constructive issues.

## Supplementary Material

Supplemental file 1

Supplemental file 2
