# Supplementary material for: Connecting the Dots between Mechanosensitive Channel Abundance, Osmotic Shock, and Survival at Single-Cell Resolution
Source: J Bacteriol. 2018 Nov 6;200(23):e00460-18. doi: 10.1128/JB.00460-18 (PMC6222198; doi:10.1128/JB.00460-18)
Supplement: Supplemental file 1 [file zjb999094913s1.pdf]

Supplemental information for “Connecting the dots between mechanosensitive channel abundance, osmotic shock, and survival at single-cell resolution”

Griffin Chure[a,†], Heun Jin Lee[b,†], Akiko Rasmussen[c], and Rob Phillips[a,b,*]

[a]Department of Biology and Biological Engineering, California Institute of Technology, Pasadena, CA, USA

[b]Department of Applied Physics, California Institute of Technology, Pasadena, CA, USA

[c]School of Medicine, Medical Sciences and Nutrition, Institute of Medical Sciences, University of Aberdeen, Foresterhill, Aberdeen AB25 2ZD United Kingdom

† contributed equally

* Send correspondence to `phillips@pboc.caltech.edu`

Supplement A: Experimental validation of MscL-sfGFP

Despite revolutionizing modern cell biology, tagging proteins with fluorophores can lead to myriad deleterious effects such as mislocalization, abrogation of functionality, or even cytotoxicity. In this section, we examine the stability and functionality of the MscL-sfGFP construct used in this work.

Comparing functionality of wild-type and fluorescently tagged MscL

To quantitatively compare the functionality between the wild-type MscL and MscL-sfGFP, patch-clamp electrophysiology experiments were conducted on each channel. Patch-clamp recordings were performed on membrane patches derived from giant protoplasts which were prepared as previously described (1). In brief, cells were grown in Luria-Bertani (LB) medium with 0.06 mg/ml cephalexin for 2.5 hours. The elongated cells were then collected by centrifugation and digested by 0.2 mg/ml lysozyme to form giant protoplasts.

Excised, inside-out patches were analyzed at a membrane potential of -20 mV with pipette and bath solutions containing 200 mM KCl, 90 mM $MgCl_2$, 10 mM $CaCl_2$, and 5 mM HEPES buffer at pH 7. All data were acquired at a sampling rate of 50 kHz with 5 kHz filtration using an AxoPatch 200B amplifier and pClamp software (Molecular Devices). The pressure threshold for activation a single MscS channel (blue stripe in Fig. S1) was compared to that of single MscL channels (yellow strip in Fig. S1). The pressure threshold for activation of the MscL channels was referenced against the

activation threshold of MscS to determine the pressure ratio (PL:PS) for gating as previously described (2). Recordings of the transmembrane current were made of three individual patches with an average PL:PS ratio of 1.56 for MscL-sfGFP. This ratio quantitatively agrees with the PL:PS ratio of 1.54 measured in a strain (MJF429 from the Booth laboratory) which expresses the wild-type MscL protein from the chromosome. The average transient current change from MscL openings (Fig. S1 shaded yellow region) is 75 pA, corresponding to a single channel conductance of 3.7 nS, comparable to the reported values of wild-type MscL. The agreement between these two strains indicates that there is negligible difference in functionality between MscL and MscL-sfGFP, allowing us to make physiological conclusions of the wild-type channel from our experiments.

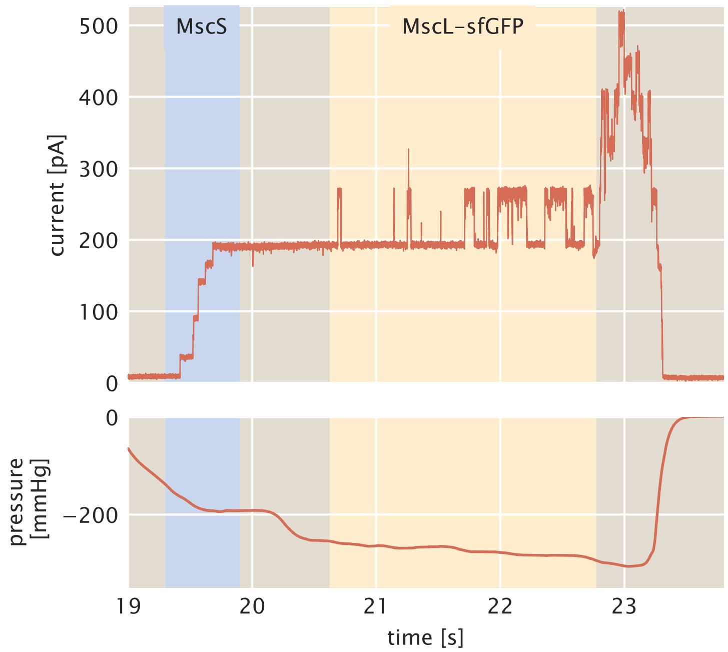

FIG S1 Characteristic MscL-sfGFP conductance obtained through patch-clamp electrophysiology. Top panel presents a characteristic measurement of channel current obtained through a patch-clamp electrophysiology measurement of bacterial protoplasts. The bottom panel shows the applied pressure through the micropipette to facilitate opening of the mechanosensitive channels. The blue shaded region indicates opening of the mechanosensitive channel of small conductance (MscS). The shaded yellow region represents opening of single MscL channels. These regions were used to compute the PL:PS ratio.

Maturation time of MscL-sfGFP

Reliable quantification of the channel copy number is paramount to this work. As such, it is important to verify that the detected fluorescence per cell accurately represents the total cellular MscL

copy number. We have made the assumption that the total fluorescence per represents all MscL-sfGFP channels present. However, it is possible that there are more channels present per cell but are not detected as the fluorophores have not properly matured. This potential error becomes more significant with longer maturation times of the fluorophore as the mean expression level changes with the growth phase of the culture. With a maturation time much longer than the typical cell division time, it is possible that the measured channel copy number represents only a fraction of the total number inherited over generations.

In our earlier work, we quantified the MscL-sfGFP channel copy number using fluorescence microscopy as well as with quantitative Western blotting. We found that these two methods agreed within 20% of the mean value, often with the counts resulting from microscopy being slightly larger than those measured through Western blotting (3). This strongly suggests that a negligible amount of channels are not observed due to inactive fluorophores.

Despite these suggestive data, we directly measured the maturation time of the superfolder GFP protein. We constructed a chromosomal integration of sfGFP expressed from a promoter under regulation from plasmid-borne TetR (*E. coli* MG1655 K12 Δ*lacIZYA ybcN::sfGFP*) . These cells were allowed to grow in LB supplemented with 500 mM NaCl held at 37°C to an OD_{600nm} of approximately 0.3. At this time, transcription and translation of the sfGFP gene was induced by addition of 10 ng/mL of anhydrous tetracycline. This expression was allowed to occur for three minutes before the addition of 100 µg/mL of kanamycin, ceasing proper protein synthesis. Three minutes of expression was chosen to provide enough time for transcription and translation. The sfGFP variant used in this work is 1155 base pairs. We can assume that the rate for transcription is 42 nucleotides per second (BNID 108488)(4), meaning approximately 28 seconds are needed to transcribe the gene. The translation rate is on the order of 10 amino acids per second, (12 - 42 amino acids / s, BNID 100059)(4). This means that 39 seconds are needed to complete translation. In total, approximately one minute is needed to complete expression of the genes. These numbers are not known for LB supplemented with 500 mM NaCl but may be reduced. For this reason, we extended the length of induction to three minutes before translation was ceased.

The excess anhydrous tetracycline was removed from the culture through centrifugation and washing with one volume of LB supplemented with 500 mM NaCl and 100 µg/mL kanamycin at 37°C. The maturation of sfGFP was then monitored through flow cytometry by measuring the mean expression of 100,000 cells every 60 to 90 seconds. The result of these measurements are shown in Fig. S2.

We observe complete maturation of the protein within 20 minutes after translation of the sfGFP gene was ceased. While the growth rate in LB + 500mM NaCl varies depending on the expression of MscL-sfGFP, we typically observe doubling times between 30 and 40 minutes, as indicated by a yellow stripe in Fig. S2A. To examine the "best case" scenario for cell growth in this medium, we measured the

growth rate of the same *E. coli* strain used to measure the fluorophore maturation time (Fig. S2 B). We observed a doubling time of 35 ± 1 min, which falls in the middle of the yellow stripe shown in Fig. S2 A. These data, coupled with our previous quantification of MscL copy number using independent methods, suggests that the fluorescence measurements made in this work reflect the total amount of MscL protein expressed.

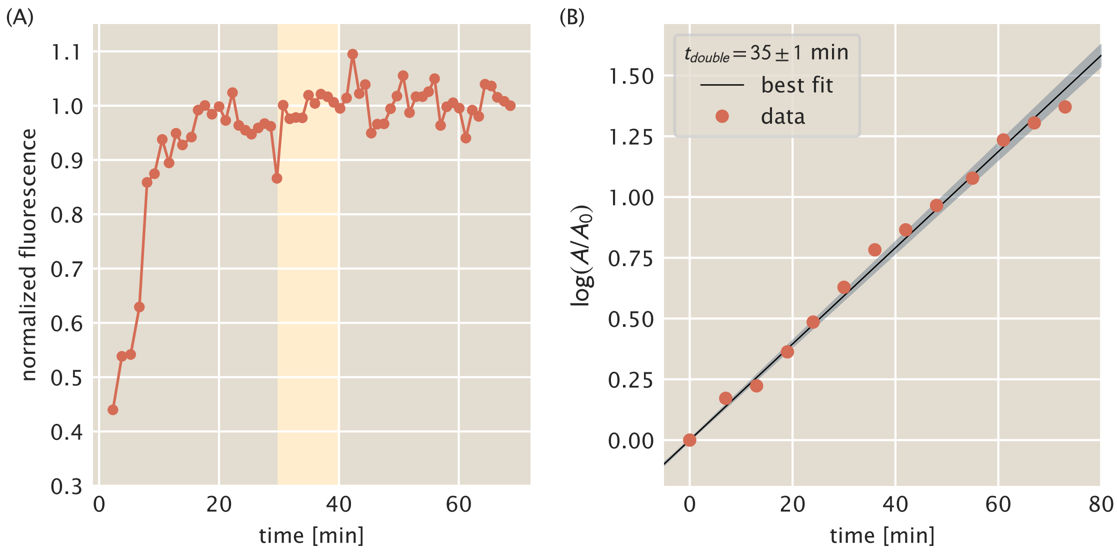

FIG S2 Measurement of sfGFP maturation as a function of time through flow cytometry. (A) Measurement of sfGFP fluorescence intensity as a function of time after cessation of protein translation. Points and connected lines indicate means of gated flow cytometry intensity distributions. Yellow stripe indicates the range of doubling times observed for the various RBS mutant strains described in this work (B) Growth curve of *E. coli* MG1655 cells in LB + 500mM NaCl. Red points indicate individual absorbance measurements. Line of best fit is shown in black with the uncertainty shown in shaded gray. The measured doubling time was 35 ± 1 min.

Quantifying Presence of MscL-sfGFP aggregates

van den berg et al. (2016) (5) showed that high levels of expression of MscL-mEos3.2 resulted in aggregates of channels, altering the physiology. To ensure that our method of measurement does not compromise our ability to draw physiological conclusions, it is important to quantify the extent of this phenomenon in our data as well as any bias it may impart on our analysis. We do indeed see fluorescent puncta in our data, yet it is possible these arise from simple statistical organization along the membrane. In van den Berg et al. 2016 (5), puncta were imaged using super-resolution microscopy, allowing tracking of their movement and calculation of a diffusion coefficient. Unfortunately, our

data is limited to single snapshots, prohibiting us from using diffusion as an identifying property of aggregation. We are therefore restricted to using statistical measures to characterize their abundance in the data.

To quantify the abundance of puncta, we analyzed a set of images from our highest expressing Shine-Dalgarno mutant (SD1) along with one of our lowest (SD4). Rather than just quantifying the mean pixel intensity of each cell, we calculated the coefficient of variation in intensity which serves as a measure of spatial uniformity. If the fluorescent proteins were very well behaved and showed no aggregation, one would näively expect the variation in intensity of the cell to be small with the intensity being relatively uniform across the entire segmented area. However, if there were aggregates, one would observe the formation of fluorescent puncta which would result in larger variation. In our reanalysis, we found that a very small proportion of cells of our highest expressing strain showed a large degree of variation in intensity (Fig. S3). Inspection of these images revealed that there were apparent fluorescent puncta which could be aggregates of the sfGFP tagged MscL proteins. These cells constitute approximately 10% of the total data set.

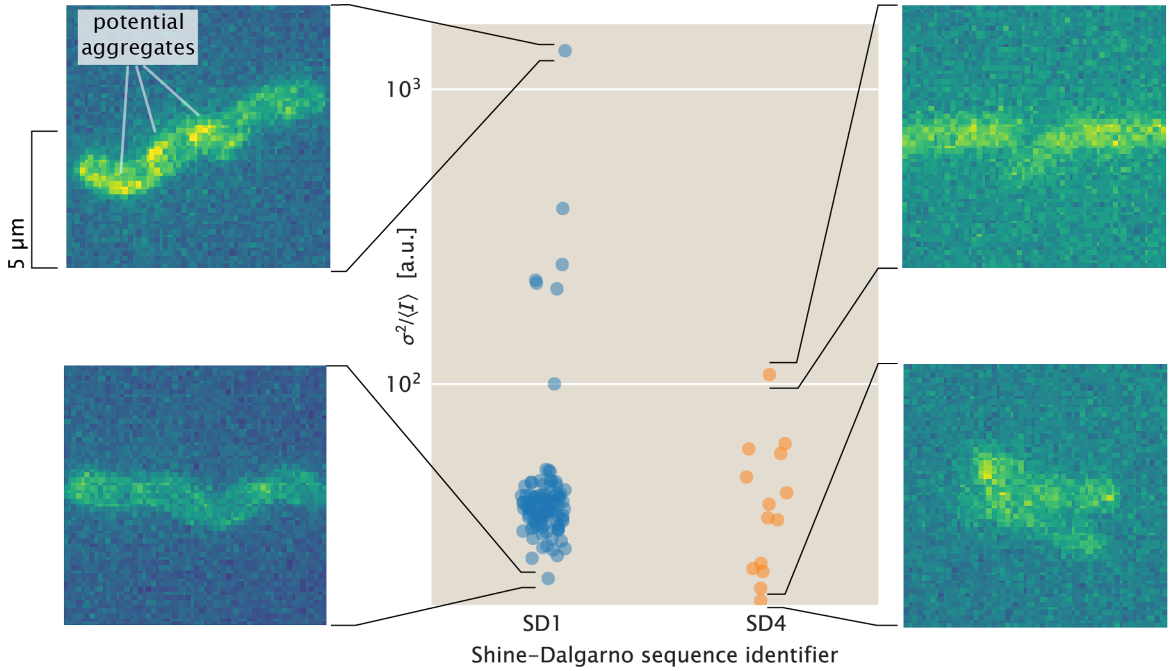

FIG S3 Distribution of potential channel aggregates under high and low expression. The noise of the measured pixel intensity is plotted with respect to the Shine-Dalgarno sequence modification. Example images are shown in false-color and are linked to their corresponding noise measurements with thin black lines. Color scale is relative to each image and cannot be compared between cells.

However, it is possible that the observed puncta are not aggregates but are rather the result of density fluctuations, where several channels happen to diffuse within a distance comparable to diffraction limited spot. We can test if this null hypothesis could explain our observation by making a simple stochastic model. Any channels within about 250 nm of each other would appear as a single fluorescent spot. We can make a simple estimate of the likelihood of observing a given number of MscL channels in a diffraction limited spot by coarse graining the cell membrane into 250 nm by 250 nm bins, as is shown in Fig. S4A. Suppose that we have a 4 μm^2 sheet of cell membrane (an area similar to that observed in our experiments) split into 64 boxes each 250 nm on a side. Assuming that the closed MscL channel is approximately 10 nm in diameter, up to 625 pentameric channels can theoretically fit in one of these lattice sites. For our purposes it is fair to assume as null hypothesis that each lattice site has an equal probability of being occupied by an MscL channel. Using the mean expression value of our highest expressing strain (500 channels per cell), we can compute the probability distribution for number of MscL channels per lattice site, as is shown in Fig. S4B. We would expect to find seven MscL channels on average per site, which would all appear to be within the same diffraction limited spot. From our data, we find that on average there are 17 MscL channels per punctum, constituting approximately 3% of the total cellular channel copy number (Fig. S4C). The probability distribution shown in Fig. S4B predicts that approximately 5% of the lattice sites will have 15 or more MscL channels, which agrees with our experimental measurement of 3%. It is therefore unclear whether the observed puncta in the high-expressing cells are the result of aggregation of protein or merely a consequence of the statistics of partitioning.

Regardless, these cells rarely appear in our data, suggesting that any pathological consequences of punctate cells bear little weight in our conclusions regarding channel abundance dependent survival. The electrophysiology trace shown in Fig. S1 suggests that sfGFP tagged channels function identically to the wild-type untagged version in terms of conductance and gating tension (6). It has been previously shown that even wild-type MscL can form clusters in reconstituted membranes which can result in a hampered gating tension, although van den Berg et al. (5) propose their data does not suggest such cluster formation. It is therefore plausible that if the putative puncta observed in our data are aggregates, there may be none to little consequence when it comes to surviving an osmotic shock.

Supplement B: Standard Candle Calibration

To estimate the single-cell MscL abundance via microscopy, we needed to determine a calibration factor that could translate arbitrary fluorescence units to protein copy number. To compute this calibration factor, we relied on *a priori* knowledge of the mean copy number of MscL-sfGFP for a particular bacterial strain in specific growth conditions. In Bialecka-Fornal et al. 2012 (3), the average MscL copy number

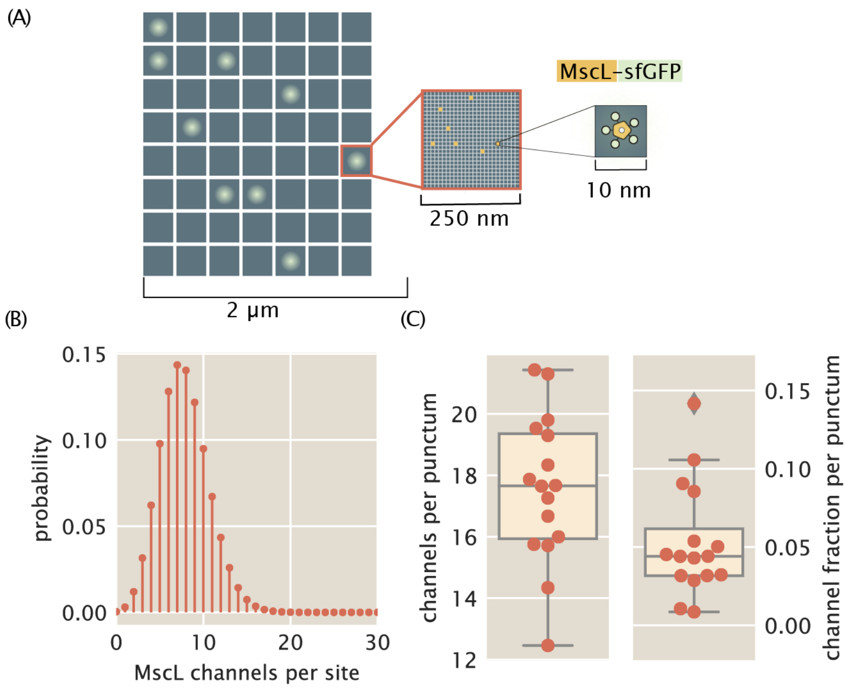

FIG S4 Formation of diffraction limited puncta from statistical positioning of MscL channels. (A) A 4µm^2 sheet of membrane split into 64 boxes, each with a 250 nm edge length. All channels within one of these boxes would appear as a single fluorescent punctum. Each box can be split into 625 individual sites with a width of 10 nm (middle), each of which can accommodate a single MscL-sfGFP pentameric channel (right). (B) Probability distribution of number of MscL channels per 250 nm edge length box. Total cellular number of channels was taken as 500. (C) Observed distribution of channels per punctum (left) and fraction of channels found in each punctum (right). Individual measurement are shown in red. The box represents the interquartile region, centerline corresponds to the median, and whiskers extend to 1.5 times the maximum and minimum interquartile region.

for a population of cells expressing an MscL-sfGFP fusion (*E. coli* K-12 MG1655 ϕ(*mscL-sfGFP*)) cells was measured using quantitative Western blotting and single-molecule photobleaching assays. By growing this strain in identical growth and imaging conditions, we can make an approximate measure of this calibration factor. In this section, we derive a statistical model for estimating the most-likely value of this calibration factor and its associated error.

Definition of a calibration factor

We assume that all detected fluorescence signal from a particular cell is derived from the MscL-sfGFP protein, after background subtraction and correction for autofluorescence. The arbitrary units of fluorescence can be directly related to the protein copy number via a calibration factor α,

$$I_\text{tot} = \alpha N_\text{tot}, \tag{1}$$

where I_tot is the total cell fluorescence and N_tot is the total number of MscL proteins per cell. Bialecka-Fornal et al. report the average cell Mscl copy number for the population rather than the distribution. Knowing only the mean, we can rewrite Eq. 1 as

$$\langle I_\text{tot} \rangle = \alpha \langle N_\text{tot} \rangle, \tag{2}$$

assuming that α is a constant value that does not change from cell to cell or fluorophore to fluorophore.

The experiments presented in this work were performed using non-synchronously growing cultures. As there is a uniform distribution of growth phases in the culture, the cell size distribution is broad the the extremes being small, newborn cells and large cells in the process of division. As described in the main text, the cell size distribution of a population is broadened further by modulating the MscL copy number with low copy numbers resulting in aberrant cell morphology. To speak in the terms of an effective channel copy number, we relate the average areal intensity of the population to the average cell size,

$$\langle I_\text{tot} \rangle = \langle I_A \rangle \langle A \rangle = \alpha \langle N_\text{tot} \rangle, \tag{3}$$

where $\langle I_A \rangle$ is the average areal intensity in arbitrary units per pixel of the population and $\langle A \rangle$ is the average area of a segmented cell. As only one focal plane was imaged in these experiments, we could not compute an appropriate volume for each cell given the highly aberrant morphology. We therefore opted to use the projected two-dimensional area of each cell as a proxy for cell size. Given this set of measurements, the calibration factor can be computed as

$$\alpha = \frac{\langle I_A \rangle \langle A \rangle}{\langle N_\text{tot} \rangle}. \tag{4}$$

While it is tempting to use Eq. 4 directly, there are multiple sources of error that are important to propagate through the final calculation. The most obvious error to include is the measurement error

reported in Bialecka-Fornal et al. 2012 for the average MscL channel count (3). There are also slight variations in expression across biological replicates that arise from a myriad of day-to-day differences. Rather than abstracting all sources of error away into a systematic error budget, we used an inferential model derived from Bayes' theorem that allows for the computation of the probability distribution of α.

Estimation of α for a single biological replicate

A single data set consists of several hundred single-cell measurements of intensity, area of the segmentation mask, and other morphological quantities. The areal density I_A is computed by dividing the total cell fluorescence by the cell area A. We are interested in computing the probability distributions for the calibration factor α, the average cell area $\langle A \rangle$, and the mean number of channels per cell $\langle N_{\text{tot}} \rangle$ for the data set as a whole given only I_A and A. Using Bayes' theorem, the probability distribution for these parameters given a single cell measurement, hereafter called the posterior distribution, can be written as

$$g(\alpha, \langle A \rangle, \langle N_{\text{tot}} \rangle \mid A, I_A) = \frac{f(A, I_A \mid \alpha, \langle A \rangle, \langle N_{\text{tot}} \rangle) g(\alpha, \langle A \rangle, \langle N_{\text{tot}} \rangle)}{f(\alpha, I_A)}, \tag{5}$$

where g and f represent probability density functions over parameters and data, respectively. The term $f(A, I_A \mid \alpha, \langle A \rangle, \langle N_{\text{tot}} \rangle)$ in the numerator represents the likelihood of observing the areal intensity I_A and area A of a cell for a given values of α, $\langle A \rangle$, and $\langle N_{\text{tot}} \rangle$. The second term in the numerator $g(\alpha, \langle A \rangle, \langle N_{\text{tot}} \rangle)$ captures all prior knowledge we have regarding the possible values of these parameters knowing nothing about the measured data. The denominator, $f(I_A, A)$ captures the probability of observing the data knowing nothing about the parameter values. This term, in our case, serves simply as a normalization constant and is neglected for the remainder of this section.

To determine the appropriate functional form for the likelihood and prior, we must make some assumptions regarding the biological processes that generate them. As there are many independent processes that regulate the timing of cell division and cell growth, such as DNA replication and peptidoglycan synthesis, it is reasonable to assume that for a given culture the distribution of cell size would be normally distributed with a mean of $\langle A \rangle$ and a variance $\sigma_{\langle A \rangle}$. Mathematically, we can write this as

$$f(A \mid \langle A \rangle, \sigma_{\langle A \rangle}) \propto \frac{1}{\sigma_{\langle A \rangle}} \exp\left[-\frac{(A - \langle A \rangle)^2}{2\sigma_{\langle A \rangle}^2}\right], \tag{6}$$

where the proportionality results from dropping normalization constants for notational simplicity.

While total cell intensity is intrinsically dependent on the cell area the areal intensity I_A is independent of cell size. The myriad processes leading to the detected fluorescence, such as translation and proper protein folding, are largely independent, allowing us to assume a normal distribution for I_A as well with a mean $\langle I_A \rangle$ and a variance $\sigma_{I_A}^2$. However, we do not have knowledge of the average areal intensity

for the standard candle strain *a priori*. This can be calculated knowing the calibration factor, total MscL channel copy number, and the average cell area as

$$I_A = \frac{\alpha \langle N_{\text{tot}} \rangle}{\langle A \rangle}. \tag{7}$$

Using Eq. 7 to calculate the expected areal intensity for the population, we can write the likelihood as a Gaussian distribution,

$$f(I_A \,|\, \alpha, \langle A \rangle, \langle N_{\text{tot}} \rangle, \sigma_{I_A}) \propto \frac{1}{\sigma_{I_A}} \exp\left[-\frac{\left(I_A - \frac{\alpha \langle N_{\text{tot}} \rangle}{\langle A \rangle}\right)^2}{2\sigma_{I_A}^2}\right]. \tag{8}$$

With these two likelihoods in hand, we are tasked with determining the appropriate priors. As we have assumed normal distributions for the likelihoods of $\langle A \rangle$ and I_A, we have included two additional parameters, $\sigma_{\langle A \rangle}$ and σ_{I_A}, each requiring their own prior probability distribution. It is common practice to assume maximum ignorance for these variances and use a Jeffreys prior (7),

$$g(\sigma_{\langle A \rangle}, \sigma_{I_A}) = \frac{1}{\sigma_{\langle A \rangle} \sigma_{I_A}}. \tag{9}$$

The next obvious prior to consider is for the average channel copy number $\langle N_{\text{tot}} \rangle$, which comes from Bialecka-Fornal et al. 2012. In this work, they report a mean μ_N and variance σ_N^2, allowing us to assume a normal distribution for the prior,

$$g(\langle N_{\text{tot}} \rangle \,|\, \mu_N, \sigma_N) \propto \frac{1}{\sigma_N} \exp\left[-\frac{(\langle N_{\text{tot}} \rangle - \mu_N)^2}{2\sigma_N^2}\right]. \tag{10}$$

For α and $\langle A \rangle$, we have some knowledge of what these parameters can and cannot be. For example, we know that neither of these parameters can be negative. As we have been careful to not overexpose the microscopy images, we can say that the maximum value of α would be the bit-depth of our camera. Similarly, it is impossible to segment a single cell with an area larger than our camera's field of view (although there are biological limitations to size below this extreme). To remain maximally uninformative, we can assume that the parameter values are uniformly distributed between these bounds, allowing us to state

$$g(\alpha) = \begin{cases} \frac{1}{\alpha_{\text{max}} - \alpha_{\text{min}}} & \alpha_{\text{min}} \leq \alpha \leq \alpha_{\text{max}} \\ 0 & \text{otherwise} \end{cases}, \tag{11}$$

for α and

$$g(\langle A \rangle) = \begin{cases} \frac{1}{\langle A \rangle_{\text{max}} - \langle A \rangle_{\text{min}}} & \langle A \rangle_{\text{min}} \leq \langle A \rangle \leq \langle A \rangle_{\text{max}} \\ 0 & \text{otherwise} \end{cases} \tag{12}$$

for $\langle A \rangle$.

Piecing Eq. 6 through Eq. 12 together generates a complete posterior probability distribution for the parameters given a single cell measurement. This can be generalized to a set of k single cell measurements as

$$g(\alpha, \langle A \rangle, \langle N_{\text{tot}} \rangle, \sigma_{I_A}, \sigma_{\langle A \rangle} \mid [I_A, A], \mu_N, \sigma_N) \propto \frac{1}{(\alpha_{\text{max}} - \alpha_{\text{min}})(\langle A \rangle_{\text{max}} - \langle A \rangle_{\text{min}})} \frac{1}{(\sigma_{I_A}\sigma_{\langle A \rangle})^{k+1}} \times \frac{1}{\sigma_N} \exp\left[-\frac{(\langle N_{\text{tot}} \rangle - \mu_N)^2}{2\sigma_N^2}\right] \prod_i^k \exp\left[-\frac{(A^{(i)} - \langle A \rangle)^2}{2\sigma_{\langle A \rangle}^2} - \frac{\left(I_A^{(i)} - \frac{\alpha \langle N_{\text{tot}} \rangle}{\langle A \rangle}\right)^2}{2\sigma_{I_A}^2}\right], \tag{13}$$

where $[I_A, A]$ represents the set of k single-cell measurements.

As small variations in the day-to-day details of cell growth and sample preparation can alter the final channel count of the standard candle strain, it is imperative to perform more than a single biological replicate. However, properly propagating the error across replicates is non trivial. One option would be to pool together all measurements of n biological replicates and evaluate the posterior given in Eq. 13. However, this by definition assumes that there is no difference between replicates. Another option would be to perform this analysis on each biological replicate individually and then compute a mean and standard deviation of the resulting most-likely parameter estimates for α and $\langle A \rangle$. While this is a better approach than simply pooling all data together, it suffers a bias from giving each replicate equal weight, skewing the estimate of the most-likely parameter value if one replicate is markedly brighter or dimmer than the others. Given this type of data and a limited number of biological replicates ($n = 6$ in this work), we chose to extend the Bayesian analysis presented in this section to model the posterior probability distribution for α and $\langle A \rangle$ as a hierarchical process in which α and $\langle A \rangle$ for each replicate is drawn from the same distribution.

A hierarchical model for estimating α

In the previous section, we assumed maximally uninformative priors for the most-likely values of α and $\langle A \rangle$. While this is a fair approach to take, we are not completely ignorant with regard to how these values are distributed across biological replicates. A major assumption of our model is that the most-likely value of α and $\langle A \rangle$ for each biological replicate are comparable, so long as the experimental error between them is minimized. In other words, we are assuming that the most-likely value for each parameter for each replicate is drawn from the same distribution. While each replicate may have a unique value, they are all related to one another. Unfortunately, proper sampling of this distribution requires an extensive amount of experimental work, making inferential approaches more attractive.

This approach, often called a multi-level or hierarchical model, is schematized in Fig. S5. Here, we use an informative prior for α and $\langle A \rangle$ for each biological replicate. This informative prior probability distribution can be described by summary statistics, often called hyper-parameters, which are then

treated as the "true" value and are used to calculate the channel copy number. As this approach allows us to get a picture of the probability distribution of the hyper-parameters, we are able to report a point estimate for the most-likely value along with an error estimate that captures all known sources of variation.

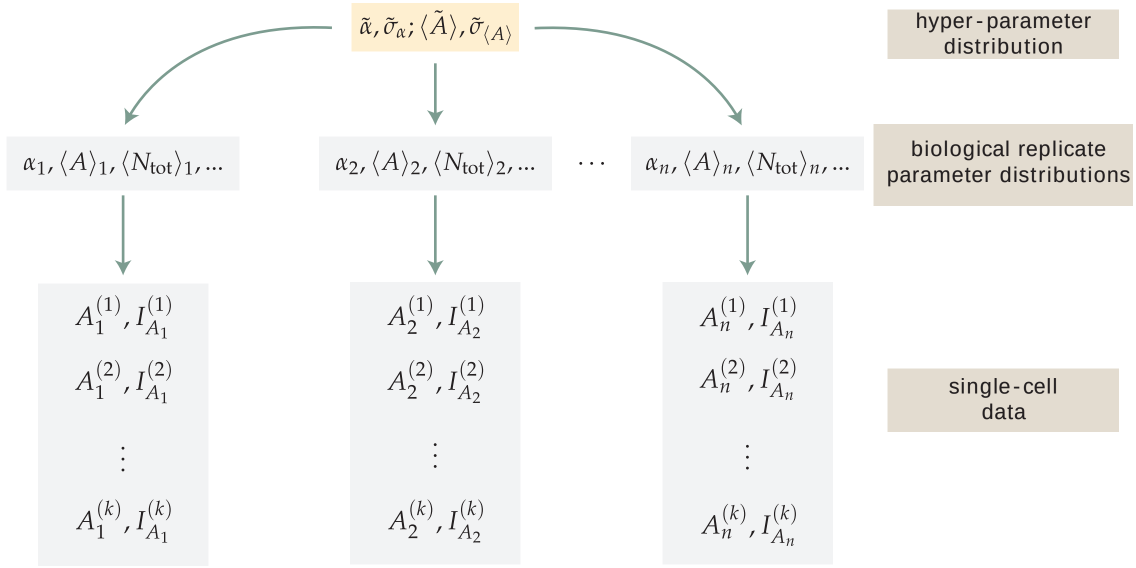

FIG S5 Schematic of hierarchical model structure. The hyper-parameter probability distributions (top panel) are used as an informative prior for the most-likely parameter values for each biological replicate (middle panel). The single-cell measurements of cell area and areal intensity (bottom panel) are used as data in the evaluation of the likelihood.

The choice for the functional form for the informative prior is often not obvious and can require other experimental approaches or back-of-the-envelope estimates to approximate. Each experiment in this work was carefully constructed to minimize the day-to-day variation. This involved adhering to well-controlled growth temperatures and media composition, harvesting of cells at comparable optical densities, and ensuring identical imaging parameters. As the experimental variation is minimized, we can use our knowledge of the underlying biological processes to guess at the approximate functional form. For similar reasons presented in the previous section, cell size is controlled by a myriad of independent processes. As each replicate is independent of another, it is reasonable to assume a normal distribution for the average cell area for each replicate. This normal distribution is described by a mean $\langle\tilde{A}\rangle$ and variance $\tilde{\sigma}_{\langle A\rangle}$. Therefore, the prior for $\langle A\rangle$ for n biological replicates can be written as

$$g(\langle A\rangle \mid \langle\tilde{A}\rangle, \tilde{\sigma}_{\langle A\rangle}) \propto \frac{1}{\tilde{\sigma}^n_{\langle A\rangle}} \prod_{j=1}^{n} \exp\left[-\frac{(\langle A\rangle_j - \langle\tilde{A}\rangle)^2}{2\tilde{\sigma}^2_{\langle A\rangle}}\right]. \tag{14}$$

In a similar manner, we can assume that the calibration factor for each replicate is normally distributed

with a mean $\tilde{\alpha}$ and variance $\tilde{\sigma}_\alpha$,

$$g(\alpha \mid \tilde{\alpha}, \tilde{\sigma}_\alpha) \propto \frac{1}{\tilde{\sigma}_\alpha^n} \prod_{j=1}^{n} \exp\left[-\frac{(\alpha_j - \tilde{\alpha})^2}{2\tilde{\sigma}_\alpha^2}\right]. \tag{15}$$

With the inclusion of two more normal distributions, we have introduced four new parameters, each of which needing their own prior. However, our knowledge of the reasonable values for the hyper-parameters has not changed from those described for a single replicate. We can therefore use the same maximally uninformative Jeffreys priors given in Eq. 9 for the variances and the uniform distributions given in Eq. 11 and Eq. 12 for the means. Stitching all of this work together generates the full posterior probability distribution for the best-estimate of $\tilde{\alpha}$ and $\langle \tilde{A} \rangle$ shown in Eq. 2 given n replicates of k single cell measurements,

$$\begin{aligned} g(\tilde{\alpha}, \tilde{\sigma}_\alpha, \langle \tilde{A} \rangle, \tilde{\sigma}_{\langle A \rangle}, \{\langle N_\text{tot} \rangle, \langle A \rangle, \alpha, \sigma_{I_A}\} \mid [I_A, A], \mu_N, \sigma_N) \propto \\ \frac{1}{(\tilde{\alpha}_\text{max} - \tilde{\alpha}_\text{min})(\langle \tilde{A} \rangle_\text{max} - \langle \tilde{A} \rangle_\text{min})\sigma_N^n (\tilde{\sigma}_\alpha \tilde{\sigma}_{\langle A \rangle})^{n+1}} \times \\ \prod_{j=1}^{n} \exp\left[-\frac{(\langle N \rangle_j^{(i)} - \mu_N)^2}{2\sigma_N^2} - \frac{(\alpha_j - \tilde{\alpha})^2}{2\tilde{\sigma}_\alpha^2} - \frac{(\langle A \rangle_j - \langle \tilde{A} \rangle)^2}{2\tilde{\sigma}_{\langle A \rangle}^2}\right] \times \\ \frac{1}{(\sigma_{I_{Aj}} \sigma_{\langle A \rangle_j})^{k_j+1}} \prod_{i=1}^{k_j} \exp\left[-\frac{(A_j^{(i)} - \langle A \rangle_j)^2}{2\sigma_{\langle A \rangle_j}^{(i)2}} - \frac{\left(I_{Aj}^{(i)} - \frac{\alpha_j \langle N_\text{tot} \rangle_j}{\langle A \rangle_j}\right)^2}{2\sigma_{I_{Aj}}^{(i)2}}\right] \end{aligned}, \tag{16}$$

where the braces $\{\ldots\}$ represent the set of parameters for biological replicates and the brackets $[\ldots]$ correspond to the set of single-cell measurements for a given replicate.

While Eq. 16 is not analytically solvable, it can be easily sampled using Markov chain Monte Carlo (MCMC). The results of the MCMC sampling for $\tilde{\alpha}$ and $\langle \tilde{A} \rangle$ can be seen in Fig. S6. From this approach, we found the most-likely parameter values of 3300^{+700}_{-700} a.u. per MscL channel and $5.4^{+0.4}_{-0.5}$ μm^2 for $\tilde{\alpha}$ and $\langle \tilde{A} \rangle$, respectively. Here, we've reported the median value of the posterior distribution for each parameter with the upper and lower bound of the 95% credible region as superscript and subscript, respectively. These values and associated errors were used in the calculation of channel copy number.

Effect of correction

The posterior distributions for α and $\langle A \rangle$ shown in Fig. S6 were used directly to compute the most-likely channel copy number for each measurement of the Shine-Dalgarno mutant strains, as is described in the coming section (*Logistic Regression*). The importance of this correction can be seen in Fig. S7. Cells with low abundance of MscL channels exhibit notable morphological defects, as illustrated in Fig. S7A. While these would all be considered single cells, the two-dimensional area of each may be comparable to two or three wild-type cells. For all of the Shine-Dalgarno mutants, the distribution of projected cell

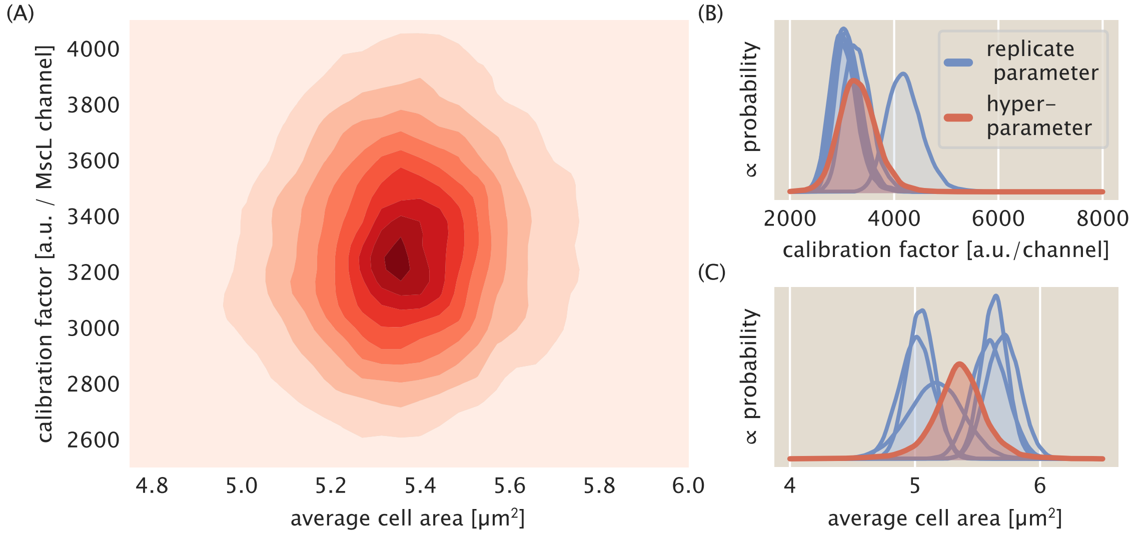

FIG S6 Posterior distributions for hyper-parameters and replicate parameters. (A) The posterior probability distribution for $\tilde{\alpha}$ and $\langle \tilde{A} \rangle$. Probability increases from light to dark red. The replicate parameter (blue) and hyper-parameter (red) marginalized posterior probability distributions for α (B) and $\langle A \rangle$ (C).

area has a long tail, with the extremes reaching 35 µm^2 per cell (Fig. S7B). Calcuating the total number of channels per cell does nothing to decouple this correlation between cell area and measured cell intensity. Fig. S7C shows the correlation between cell area and the total number of channels without normalizing to an average cell size $\langle A \rangle$ differentiated by their survival after an osmotic down-shock. This correlation is removed by calculating an effective channel copy number shown in Fig. S7D.

Choice of growth medium

To measure the calibration factor, we chose to grow the MLG910 *E. coli* strain in LB Miller medium as was performed in Bialecka-Fornal et al. 2012 (3), despite the fact that the Shine-Dalgarno sequence mutants used in this work were grown in LB supplemented with 500 mM NaCl. While a variety of growth media were tested in Bialecka-Fornal et al. 2012, the MscL-sfGFP copy number was not measured in this high-salt medium. To test if the calibration factor was dependent on the growth medium, we compared the estimated calibration factor in LB to that estimated in M9 supplemented with 0.5% glucose. These two media have starkly different compositions, serving as a good probe of the sensitivity of the calibration factor to intracellular chemistry.

E. coli strain MLG910 expressing an MscL-sfGFP fusion was grown in LB Miller, LB + 500 mM NaCl, and in M9 + 0.5% glucose to early exponential phase ($OD_{600nm} \sim 0.3$) and were imaged on a

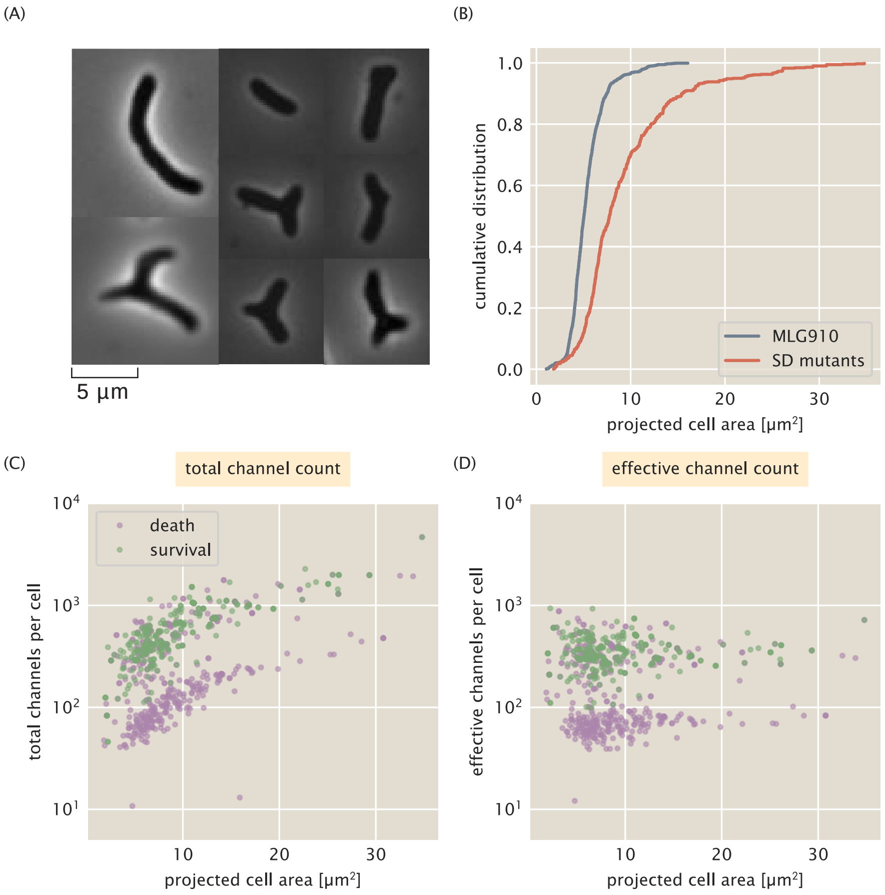

FIG S7 Influence of area correction for Shine-Dalgarno mutants. (A) Representative images of aberrant cell morphologies found in low-expressing Shine-Dalgarno mutants. (B) Empirical cumulative distribution of two-dimensional projected cell area for the standard candle strain MLG910 (gray line) and for all Shine-Dalgarno mutants (red line). (C) The correlation between channel copy number and cell area without the area correction. (D) The correlation between effective channel copy number and cell area with the area correction applied.

rigid agarose substrate. The cumulative intensity distributions for these growth media are shown in Fig. S8. There is a notable difference in the fluorescence distribution between LB Miller (blue line) and M9 + 0.5% glucose (red), as is expected given the estimated channel copy numbers reported in (3). The fluorescence distribution from MLG910 grown in LB + 500 mM NaCl (green line) is similar, yet not identical, to that grown in LB Miller broth. Using the count of 340 $\pm$ 68 and 466 $\pm$ 64 channels per cell for MLG910 grown in LB Miller and M9 + 0.5% glucose, respectively, coupled with the measurements shown in Fig. S8 we computed the posterior probability distribution of the calibration factor for each medium (Fig. S8 B). We found that these posterior distributions largely overlap, although the modes are distinctly different. We note that the calibration factor obtained from LB Miller completely overlaps that obtained from the M9 + 0.5% glucose data, suggesting that the error propagated through the final channel calculation accounts for any difference between the growth medium chosen.

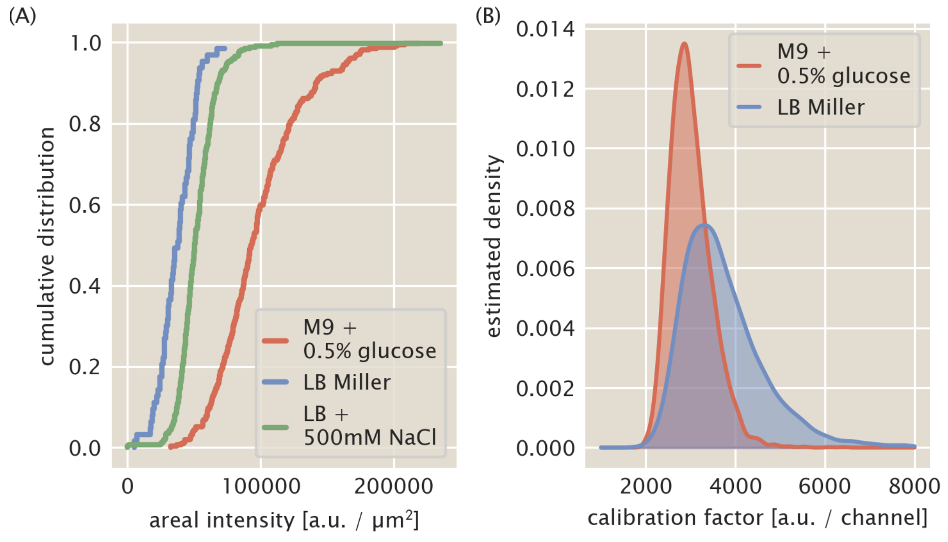

FIG S8 MLG910 intensity measurements and calibration factor estimation in different growth media. (A) Cumulative distribution of measured areal intensity (arbitrary units per square micron) measured for a collection of MLG910 cells grown in LB Miller medium (blue, medium used for calibration factor in this work), M9 + 0.5% glucose (red), and LB + 500mM NaCl (green, medium used for osmotic shock experiments). (B) Posterior probability distributions for the calibration factor (arbitrary units per MscL channel) estimated using MLG910 as a standard candle grown in LB miller (blue) and M9 + 0.5% glucose (red).

Supplement C: Classification of Cell Fate

We defined a survival event as a cell that went on to divide at least twice in the several hours following the applied osmotic shock. In nearly all of our experiments, cells which did not survive an osmotic shock exhibited necrosis with loss of phase contrast, extensive blebbing and bursting of the membrane, and the presence of dark aggregates at the cell poles. An example field across time is shown below in Fig. S9 where the cells are necrotic. We have also included a supplementary video of this field (Video S1). On occasion, we observed cells which did not obviously display the aforementioned death criteria yet did not undergo one or two division events. These cells were not counted in our experiments and were not included in the final tally of survival versus death. Across our 2822 single cell measurements, such "no call" classifications were observed only 83 times, constituting only 3% of the total cell measurements. A breakdown of all classification types and their respective abundances can be seen in Table S1.

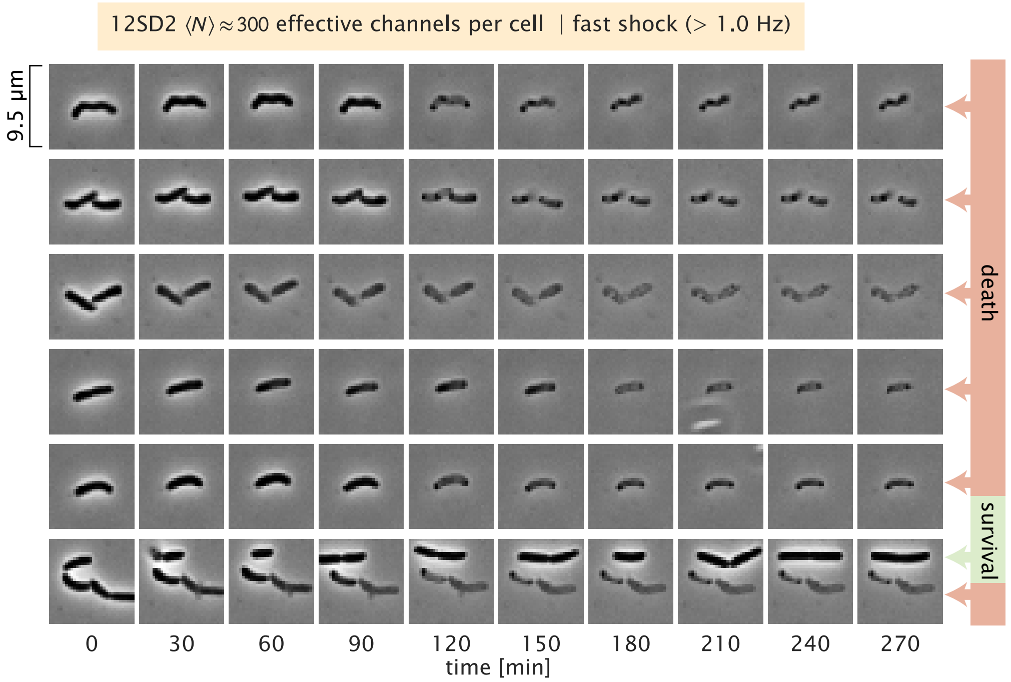

FIG S9 Time lapse of a representative field after osmotic shock and the resulting classifications. Each row shows an individual cell or pair of neighboring cells over time after the application of a fast osmotic shock. Cells classified as dead are denoted by red arrows. The lone surviving cell in this field (bottom row, top ¼ of image) is marked in green. A video file of a similar field field can be found in the supplementary material as Video S1.

TABLE S1 Cell fate classifications and their relative abundances in the complete data set. "Dead-on-Arrival" classification was assigned to cells that appeared to be dead before application of osmotic shock. "No Call" was assigned to cells to whom fate could not be applied. "Death" was assigned to cells which displayed either obvious necrosis or did not undergo at least two division events. "Survival" was assigned to cells which underwent at least two division events after osmotic shock. Neither "No Call" nor "Dead-On-Arrival" classifications were included in the final analysis.

Classification	Number of Observations	Percentage of Measurements
Dead-On-Arrival	11	0.4%
No Call	83	3%
Death	1246	44%
Survival	1482	53%

To assess the validity of our morphology-based classification scheme, we performed a subset of the osmotic shock experiments described in the manuscript using propidium iodide staining to mark cells which had compromised membranes, identifying them as dead. Briefly, cells expressing on average ~ 80 MscL channels per cell were grown in LB + 500 mM NaCl to an OD_{600nm} of approximately 0.25. The cells were then mounted in the flow cell as described in the Materials and Methods in the main text and subjected to a large osmotic shock. After the shock, the cells were monitored for two hours. The propidium iodide stain (LIVE/DEAD BacLight Bacterial Cell Viability Staining, Thermo Fisher) was then passed into the flow chamber and imaged. An example of image of the phase contrast and propidium iodide fluorescence images are shown in Fig. S10. We note that cells matching our death criteria, meaning loss of phase contrast and visible distortion of the cell membrane, were strongly marked with propidium iodide, confirming that these cells were dead. The few example of "no call" classification where survival or death could not be determined from morphology alone showed that these cells were in fact dead (see highlighted row in Fig. S10). Cells that went on to divide two or more times in this period were not significantly stained with propidium iodide, confirming their viability and effectiveness of the stain itself. Given this data set, we compared the classification breakdown using our morphology-based method with the conclusive results from the propidium iodide staining (Table S2). We found that the two approaches to defining death agreed within 1%. This agreement leads us to believe that our definition of cell survival as morphological regularity and sustained cell growth is sufficiently accurate to draw physiological conclusions from our experiments.

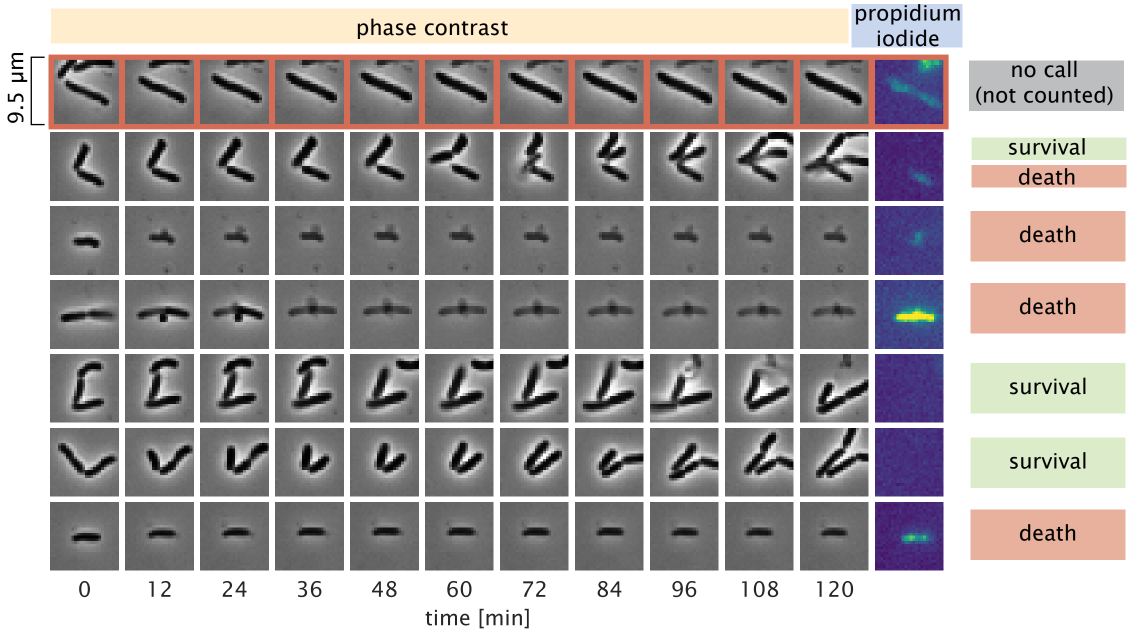

FIG S10 Representative images of propidium iodide staining after a strong osmotic shock. Phase contrast images of individual or pairs of cells as a function of time (columns). The final column corresponds to fluorescence from propidium iodide. Bright fluorescence indicates intercalation with DNA indicating cell death. Classification of survival based only from morphology is shown as text in the final column. Highlighted row indicates a "no call" event where morphology alone could not be used to determine survival or death.

TABLE S2 Comparison of morphology-based and dye-based survival classification. One cell was identified as a "No Call" in propidium iodide due to absence of propidium iodide staining despite extensive necrosis.

Classification	Observations via Morphology	Observations via Propidium Iodide Staining
Dead-On-Arrival	184	185
No Call	2	1
Survival	5	5

Supplement D: Logistic Regression

In this work, we were interested in computing the survival probability under a large hypo-osmotic shock as a function of MscL channel number. As the channel copy number distributions for each Shine-Dalgarno sequence mutant were broad and overlapping, we chose to calculate the survival probability through logistic regression – a method that requires no binning of the data providing the least biased estimate of survival probability. Logistic regression is a technique that has been used in medical statistics since the late 1950's to describe diverse phenomena such as dose response curves, criminal recidivism, and survival probabilities for patients after treatment (8–10). It has also found much use in machine learning to tune a binary or categorical response given a continuous input (11–13).

In this section, we derive a statistical model for estimating the most-likely values for the coefficients β_0 and β_1 and use Bayes' theorem to provide an interpretation for the statistical meaning.

Bayesian parameter estimation of β_0 and β_1

The central challenge of this work is to estimate the probability of survival p_s given only a measure of the total number of MscL channels in that cell. In other words, for a given measurement of N_c channels, we want to know likelihood that a cell would survive an osmotic shock. Using Bayes' theorem, we can write a statistical model for the survival probability as

$$g(p_s \mid N_c) = \frac{f(N_c \mid p_s) g(p_s)}{f(N_c)}, \tag{17}$$

where g and f represent probability density functions over parameters and data, respectively. The posterior probability distribution $g(p_s \mid N_c)$ describes the probability of p_s given a specific number of channels N_c. This distribution is dependent on the likelihood of observing N_c channels assuming a value of p_s multiplied by all prior knowledge we have about knowing nothing about the data, $g(s)$.

The denominator $f(N_c)$ in Eq. 17 captures all knowledge we have about the available values of N_c, knowing nothing about the true survival probability. As this term acts as a normalization constant, we will neglect it in the following calculations for convenience.

To begin, we must come up with a statistical model that describes the experimental measurable in our experiment – survival or death. As this is a binary response, we can consider each measurement as a Bernoulli trial with a probability of success matching our probability of survival p_s,

$$f(s \mid p_s) = p_s^s (1 - p_s)^{1-s}, \tag{18}$$

where s is the binary response of 1 or 0 for survival and death, respectively. As is stated in the introduction to this section, we decided to use a logistic function to describe the survival probability. We assume that the log-odds of survival is linear with respect to the effective channel copy number N_c as

$$\log \frac{p_s}{1 - p_s} = \beta_0 + \beta_1 N_c, \tag{19}$$

where β_0 and β_1 are coefficients which describe the survival probability in the absence of channels and the increase in log-odds of survival conveyed by a single channel. The rationale behind this interpretation is presented in the following section, *A Bayesian interpretation of β_0 and β_1*. Using this assumption, we can solve for the survival probability p_s as,

$$p_s = \frac{1}{1 + e^{-\beta_0 - \beta_1 N_c}}. \tag{20}$$

With a functional form for the survival probability, the likelihood stated in Eq. 17 can be restated as

$$f(N_c, s \mid \beta_0, \beta_1) = \left(\frac{1}{1 + e^{-\beta_0 - \beta_1 N_c}}\right)^s \left(1 - \frac{1}{1 + e^{-\beta_0 - \beta_1 N_c}}\right)^{1-s}. \tag{21}$$

As we have now introduced two parameters, β_0, and β_1, we must provide some description of our prior knowledge regarding their values. As is typically the case, we know nothing about the values for β_0 and β_1. These parameters are allowed to take any value, so long as it is a real number. Since all values are allowable, we can assume a flat distribution where any value has an equally likely probability. This value of this constant probability is not necessary for our calculation and is ignored. For a set of k single-cell measurements, we can write the posterior probability distribution stated in Eq. 17 as

$$g(\beta_0, \beta_1 \mid N_c, s) = \prod_{i=1}^{n} \left(\frac{1}{1 + e^{-\beta_0 - \beta_1 N_c^{(i)}}}\right)^{s^{(i)}} \left(1 - \frac{1}{1 + e^{-\beta_0 - \beta_1 N_c^{(i)}}}\right)^{1-s^{(i)}} \tag{22}$$

Implicitly stated in Eq. 22 is absolute knowledge of the channel copy number N_c. However, as is described in *Standard Candle Calibration*, we must convert from a measured areal sfGFP intensity I_A to a effective channel copy number,

$$N_c = \frac{I_A \langle \tilde{A} \rangle}{\tilde{\alpha}}, \tag{23}$$

where $\langle \tilde{A} \rangle$ is the average cell area of the standard candle strain and $\tilde{\alpha}$ is the most-likely value for the calibration factor between arbitrary units and protein copy number. In *Standard Candle Calibration*, we detailed a process for generating an estimate for the most-likely value of $\langle \tilde{A} \rangle$ and $\tilde{\alpha}$. Given these estimates, we can include an informative prior for each value. From the Markov chain Monte Carlo samples shown in Fig. S6, the posterior distribution for each parameter is approximately Gaussian. By approximating them as Gaussian distributions, we can assign an informative prior for each as

$$g(\alpha \mid \tilde{\alpha}, \tilde{\sigma}_\alpha) \propto \frac{1}{\tilde{\sigma}_\alpha^k} \prod_{i=1}^{k} \exp\left[-\frac{(\alpha_i - \tilde{\alpha})^2}{2\tilde{\sigma}_\alpha^2}\right] \tag{24}$$

for the calibration factor for each cell and

$$g(\langle A \rangle \mid \langle \tilde{A} \rangle, \tilde{\sigma}_{\langle A \rangle}) = \frac{1}{\tilde{\sigma}_{\langle A \rangle}^k} \prod_{i=1}^{k} \exp\left[-\frac{(\langle A \rangle_i - \langle \tilde{A} \rangle)^2}{2\tilde{\sigma}_{\langle A \rangle}^2}\right], \tag{25}$$

where $\tilde{\sigma}_\alpha$ and $\tilde{\sigma}_{\langle A \rangle}$ represent the variance from approximating each posterior as a Gaussian. The proportionality for each prior arises from the neglecting of normalization constants for notational convenience.

Given Eq. 21 through Eq. 25, the complete posterior distribution for estimating the most likely values of β_0 and β_1 can be written as

$$\begin{aligned} g(\beta_0,\beta_1 \mid [I_A, s], \langle \tilde{A} \rangle, \tilde{\sigma}_{\langle A \rangle}, \tilde{\alpha}, \tilde{\sigma}_\alpha) \propto \frac{1}{(\tilde{\sigma}_\alpha \tilde{\sigma}_{\langle A \rangle})^k} \prod_{i=1}^{k} \left(1 + \exp\left[-\beta_0 - \beta_1 \frac{I_{A_i} \langle A \rangle_i}{\alpha_i}\right]\right)^{-s_i} \times \\ \left(1 - \left(1 + \exp\left[-\beta_0 - \beta_1 \frac{I_{A_i} \langle A \rangle_i}{\alpha_i}\right]\right)^{-1}\right)^{1-s_i} \exp\left[-\frac{(\langle A \rangle_i - \langle \tilde{A} \rangle)^2}{2\tilde{\sigma}_{\langle A \rangle}} - \frac{(\alpha_i - \tilde{\alpha})^2}{2\tilde{\sigma}_\alpha^2}\right]. \end{aligned} \tag{26}$$

As this posterior distribution is not solvable analytically, we used Markov chain Monte Carlo to draw samples out of this distribution, using the log of the effective channel number as described in the main text. The posterior distributions for β_0 and β_1 for both slow and fast shock rate data can be seen in Fig. S11

A Bayesian interpretation of β_0 and β_1

The assumption of a linear relationship between the log-odds of survival and the predictor variable N_c appears to be arbitrary and is presented without justification. However, this relationship is directly connected to the manner in which Bayes' theorem updates the posterior probability distribution upon the observation of new data. In following section, we will demonstrate this connection using the relationship between survival and channel copy number. However, this description is general and can be applied to any logistic regression model so long as the response variable is binary. This connection was shown briefly by Allen Downey in 2014 and has been expanded upon in this work (14).

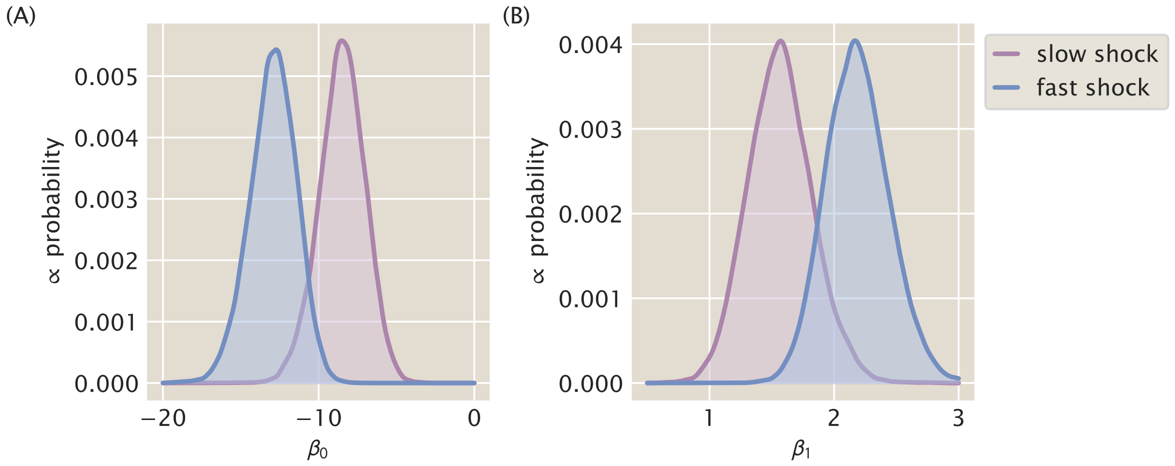

FIG S11 Posterior distributions for logistic regression coefficients evaluated for fast and slow shock rates. (A) Kernel density estimates of the posterior distribution for β_0 for fast (blue) and slow (purple) shock rates. (B) Kernel density estimates of posterior distribution for β_1.

The probability of observing a survival event s given a measurement of N_c channels can be stated using Bayes' theorem as

$$g(s \mid N_c) = \frac{f(N_c \mid s) g(s)}{f(N_c)}. \tag{27}$$

where g and f represent probability density functions over parameters and data respectively. The posterior distribution $g(s \mid N_c)$ is the quantity of interest and implicitly related to the probability of survival. The likelihood $g(N_c \mid s)$ tells us the probability of observing N_c channels in this cell given that it survives. The quantity $g(s)$ captures all *a priori* knowledge we have regarding the probability of this cell surviving and the denominator $f(N_c)$ tells us the converse – the probability of observing N_c cells irrespective of the survival outcome.

Proper calculation of Eq. 27 requires that we have knowledge of $f(N_c)$, which is difficult to estimate. While we are able to give appropriate bounds on this term, such as a requirement of positivity and some knowledge of the maximum membrane packing density, it is not so obvious to determine the distribution between these bounds. Given this difficulty, it's easier to compute the odds of survival $\mathcal{O}(s \mid N_c)$, the probability of survival s relative to death d,

$$\mathcal{O}(s \mid N_c) = \frac{g(s \mid N_c)}{g(d \mid N_c)} = \frac{f(N_c \mid s) g(s)}{f(N_c \mid d) g(d)}, \tag{28}$$

where $f(N_c)$ is cancelled. The only stipulation on the possible value of the odds is that it must be a positive value. As we would like to equally weigh odds less than one as those of several hundred or thousand, it is more convenient to compute the log-odds, given as

$$\log \mathcal{O}(s \mid N_c) = \log \frac{g(s)}{g(d)} + \log \frac{f(N_c \mid s)}{f(N_c \mid d)}. \tag{29}$$

Computing the log-transform reveals two interesting quantities. The first term is the ratio of the priors and tells us the *a priori* knowledge of the odds of survival irrespective of the number of channels. As we have no reason to think that survival is more likely than death, this ratio goes to unity. The second term is the log likelihood ratio and tells us how likely we are to observe a given channel copy number N_c given the cell survives relative to when it dies.

For each channel copy number, we can evaluate Eq. 29 to measure the log-odds of survival. If we start with zero channels per cell, we can write the log-odds of survival as

$$\log \mathcal{O}(s \mid N_c = 0) = \log \frac{g(s)}{g(d)} + \log \frac{f(N_c = 0 \mid s)}{f(N_c = 0 \mid d)}. \tag{30}$$

For a channel copy number of one, the odds of survival is

$$\log \mathcal{O}(s \mid N_c = 1) = \log \frac{g(s)}{g(d)} + \log \frac{f(N_c = 1 \mid s)}{f(N_c = 1 \mid d)}. \tag{31}$$

In both Eq. 30 and Eq. 31, the log of our *a priori* knowledge of survival versus death remains. The only factor that is changing is log likelihood ratio. We can be more general in our language and say that the log-odds of survival is increased by the difference in the log-odds conveyed by addition of a single channel. We can rewrite the log likelihood ratio in a more general form as

$$\log \frac{f(N_c \mid s)}{f(N_c \mid d)} = \log \frac{f(N_c = 0 \mid s)}{f(N_c = 0 \mid d)} + N_c \left[\log \frac{f(N_c = 1 \mid s)}{f(N_c = 1 \mid d)} - \log \frac{f(N_c = 0 \mid s)}{f(N_c = 0 \mid d)} \right], \tag{32}$$

where we are now only considering the case in which $N_c \in [0, 1]$. The bracketed term in Eq. 32 is the log of the odds of survival given a single channel relative to the odds of survival given no channels. Mathematically, this odds-ratio can be expressed as

$$\log \mathcal{OR}_{N_c}(s) = \log \frac{\frac{f(N_c=1 \mid s) g(s)}{f(N_c=1 \mid d) g(d)}}{\frac{f(N_c=0 \mid s) g(s)}{f(N_c=0 \mid d) g(d)}} = \log \frac{f(N_c = 1 \mid s)}{f(N_c = 1 \mid d)} - \log \frac{f(N_c = 0 \mid s)}{f(N_c = 0 \mid d)}. \tag{33}$$

Eq. 33 is mathematically equivalent to the bracketed term shown in Eq. 32.

We can now begin to staple these pieces together to arrive at an expression for the log odds of survival. Combining Eq. 32 with Eq. 29 yields

$$\log \mathcal{O}(s \mid N_c) = \log \frac{g(s)}{g(d)} + \log \frac{f(N_c = 0 \mid s)}{f(N_c = 0 \mid d)} + N_c \left[\frac{f(N_c = 1 \mid s)}{f(N_c = 1 \mid d)} - \log \frac{f(N_c = 0 \mid s)}{f(N_c = 0 \mid d)} \right]. \tag{34}$$

Using our knowledge that the bracketed term is the log odds-ratio and the first two times represents the log-odds of survival with $N_c = 0$, we conclude with

$$\log \mathcal{O}(s \mid N_c) = \log \mathcal{O}(s \mid N_c = 0) + N_c \log \mathcal{OR}_{N_c}(s). \tag{35}$$

This result can be directly compared to Eq. 1 presented in the main text,

$$\log \frac{p_s}{1 - p_s} = \beta_0 + \beta_1 N_c, \tag{36}$$

which allows for an interpretation of the seemingly arbitrary coefficients β_0 and β_1. The intercept term, β_0, captures the log-odds of survival with no MscL channels. The slope, β_1, describes the log odds-ratio of survival which a single channel relative to the odds of survival with no channels at all. While we have examined this considering only two possible channel copy numbers (1 and 0), the relationship between them is linear. We can therefore generalize this for any MscL copy number as the increase in the log-odds of survival is constant for addition of a single channel.

Other properties as predictor variables

The previous two sections discuss in detail the logic and practice behind the application of logistic regression to cell survival data using only the effective channel copy number as the predictor of survival. However, there are a variety of properties that could rightly be used as predictor variables, such as cell area and shock rate. As is stipulated in our standard candle calibration, there should be no correlation between survival and cell area. Fig. S12A and B show the logistic regression performed on the cell area. We see for both slow and fast shock groups,there is little change in survival probability with changing cell area and the wide credible regions allow for both positive and negative correlation between survival and area. The appearance of a bottle neck in the notably wide credible regions is a result of a large fraction of the measurements being tightly distributed about a mean value. Fig. S12C shows the predicted survival probability as a function of the the shock rate. There is a slight decrease in survivability as a function of increasing shock rate, however the width of the credible region allows for slightly positive or slightly negative correlation. While we have presented logistic regression in this section as a one-dimensional method, Eq. 19 can be generalized to n predictor variables x as

$$\log \frac{p_s}{1 - p_s} = \beta_0 + \sum_i^n \beta_i x_i. \tag{37}$$

Using this generalization, we can use both shock rate and the effective channel copy number as predictor variables. The resulting two-dimensional surface of survival probability is shown in Fig. S12D. As is suggested by Fig. S12C, the magnitude of change in survivability as the shock rate is increased is smaller than that along increasing channel copy number, supporting our conclusion that for MscL alone, the copy number is the most important variable in determining survival.

Supplement E: Shock Classification

Its been previously shown that the rate of hypo-osmotic shock dictates the survival probability (15). To investigate how a single channel contributes to survival, we queried survival at several shock rates with varying MscL copy number. In the main text of this work, we separated our experiments into

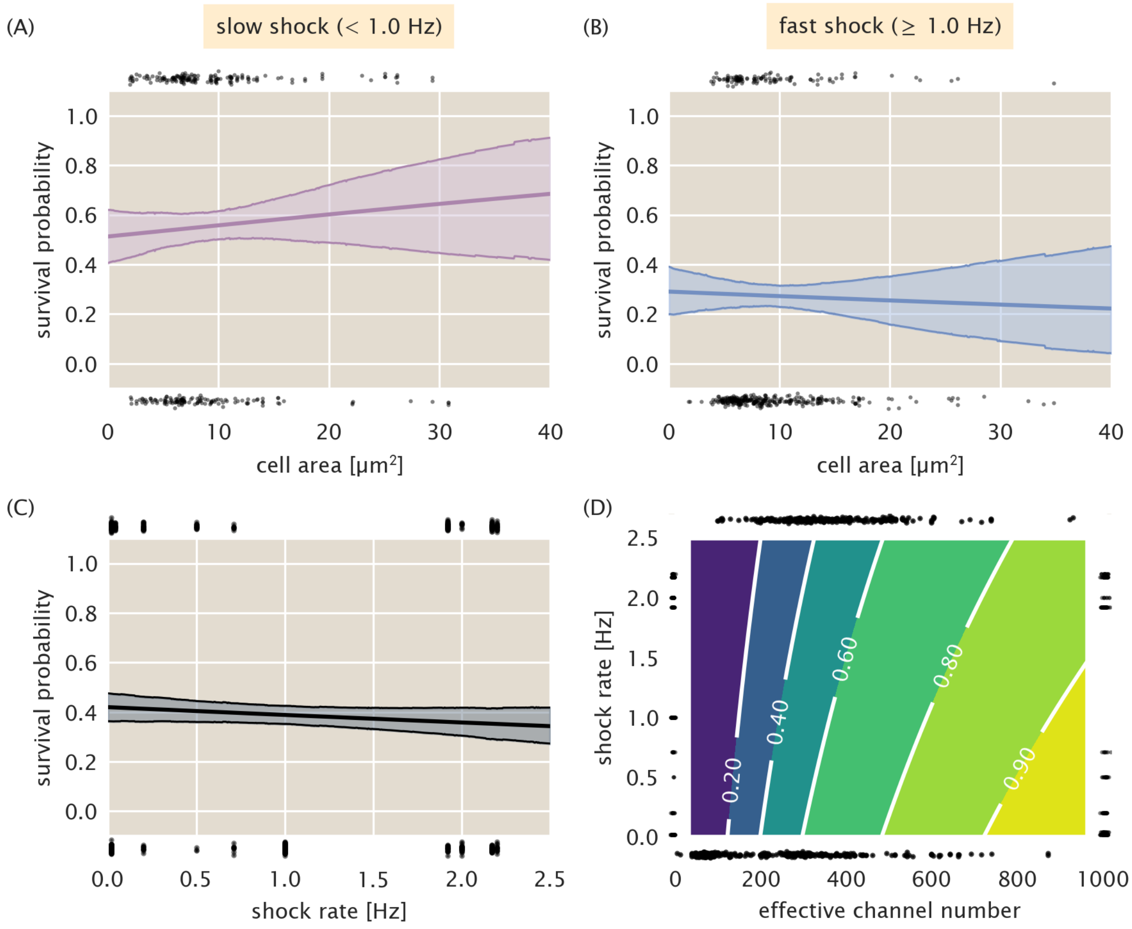

FIG S12 Survival probability estimation using alternative predictor variables. (A) Estimated survival probability as a function of cell area for the slow shock group. (B) Estimated survival probability as a function of cell area for the fast shock group. (C) Estimated survival probability as a function shock rate. Black points at top and bottom of plots represent single-cell measurements of cells who survived and perished, respectively. Shaded regions in (A) - (C) represent the 95% credible region. (D) Surface of estimated survival probability using both shock rate and effective channel number as predictor variables. Black points at left and right of plot represent single-cell measurements of cells which survived and died, respectively, sorted by shock rate. Points at top and bottom of plot represent survival and death sorted by their effective channel copy number. Labeled contours correspond to the survival probability.

arbitrary bins of "fast" ($\geq$ 1.0 Hz) and "slow" ($<$ 1.0 Hz) shock rates. In this section, we discuss our rationale for coarse graining our data into these two groupings.

As is discussed in the main text and in the supplemental section *Logistic Regression*, we used a bin-free method of estimating the survival probability given the MscL channel copy number as a predictor variable. While this method requires no binning of the data, it requires a data set that sufficiently covers the physiological range of channel copy number to accurately allow prediction of survivability. Fig. S13 shows the results of the logistic regression treating each shock rate as an individual data set. The most striking feature of the plots shown in Fig. S13 is the inconsistent behavior of the predicted survivability from shock rate to shock rate. The appearance of bottle necks in the credible regions for some shock rates (0.2Hz, 0.5Hz, 2.00Hz, and 2.20 Hz) appear due to a high density of measurements within a narrow range of the channel copy number at the narrowest point in the bottle neck. While this results in a seemingly accurate prediction of the survival probability at that point, the lack of data in other copy number regimes severely limits our extrapolation outside of the copy number range of that data set. Other shock rates (0.018 Hz, 0.04 Hz, and 1.00 Hz) demonstrate completely pathological survival probability curves due to either complete survival or complete death of the population.

Ideally, we would like to have a wide range of MscL channel copy numbers at each shock rate shown in Fig. S13. However, the low-throughput nature of these single-cell measurements prohibits completion of this within a reasonable time frame. It is also unlikely that thoroughly dissecting the shock rate dependence will change the overall finding from our work that several hundred MscL channels are needed to convey survival under hypo-osmotic stress.

Given the data shown in Fig. S13, we can try to combine the data sets into several bins. Fig. S14 shows the data presented in Fig. S13 separated into "slow" ($<$ 0.5 Hz, A), "intermediate" (0.5 - 1.0 Hz, B), and "fast" ($>$ 1.0 Hz, C) shock groups. Using these groupings, the full range of MscL channel copy numbers are covered for each case, with the intermediate shock rate sparsely sampling copy numbers greater than 200 channels per cell. In all three of these cases, the same qualitative story is told – several hundred channels per cell are necessary for an appreciable level of survival when subjected to an osmotic shock. This argument is strengthened when examining the predicted survival probability by considering all shock rates as a single group, shown in Fig. S14D. This treatment tells nearly the same quantitative and qualitative story as the three rate grouping shown in this section and the two rate grouping presented in the main text. While there does appear to be a dependence on the shock rate for survival when only MscL is expressed, the effect is relatively weak with overlapping credible regions for the logistic regression across the all curves. To account for the sparse sampling of high copy numbers observed in the intermediate shock group, we split this set and partitioned the measurements into either the "slow" ($<$ 1.0 Hz) or "fast" ($\geq$ 1.0 Hz) groups presented in the main text of this work.

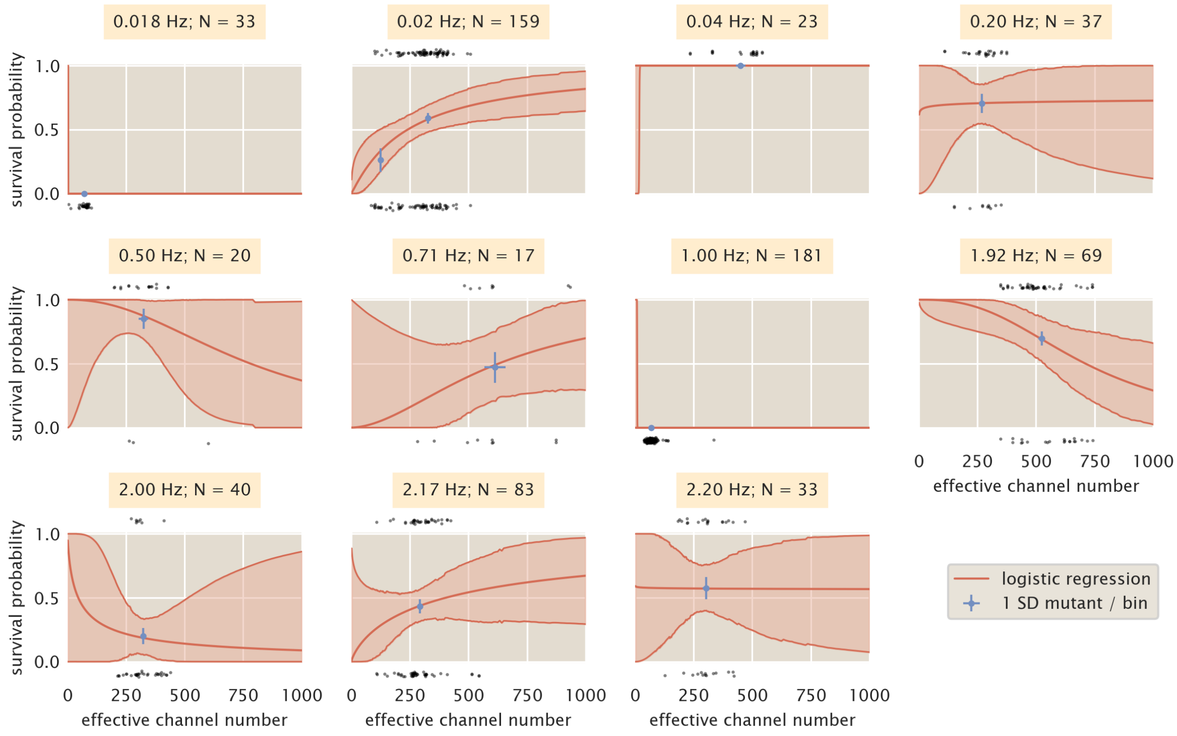

FIG S13 Binning by individual shock rates. Survival probability estimates from logistic regression (red lines) and the computed survival probability for all SD mutants subjected to that shock rate (blue points). Black points at top and bottom of each plot correspond to single cell measurements of survival (top) and death (bottom). Red shaded regions signify the 95% credible region of the logistic regression. Horizontal error bars of blue points are the standard error of the mean channel copy number. Vertical error bars of blue points correspond to the uncertainty in survival probability by observing n survival events from N single-cell measurements.

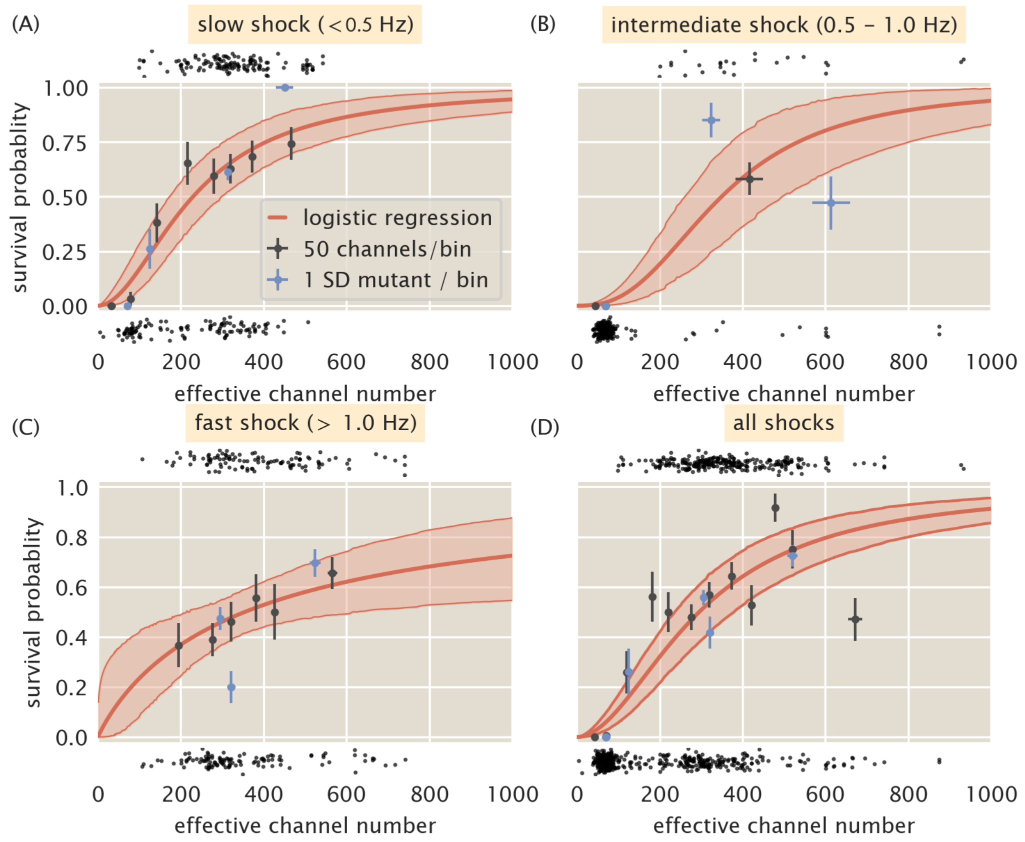

FIG S14 Coarse graining shock rates into different groups. Estimated survival probability curve for slow (A), intermediate (B), and fast (C) shock rates. (D) Estimated survival probability curve from pooling all data together, ignoring varying shock rates. Red shaded regions correspond to the 95% credible region of the survival probability estimated via logistic regression. Black points at top and bottom of each plot represent single-cell measurements of cells which survived and died, respectively. Black points and error bars represent survival probability calculations from bins of 50 channels per cell. Blue points represent the survival probability for a given Shine-Dalgarno mutant. Horizontal error bars are the standard error of the mean with at least 25 measurements and vertical error bars signifies the uncertainty in the survival probability from observing n survival events out of N total measurements.

Supplement F: Comparison of Survival Probability with van den Berg et al. 2016

In van den Berg et al. 2016, the authors report a 100% survival rate at approximately 100 channels per cell. While the number of mechanosensitive channels per cell was quantified at the level of single cells, the survival probability was measured in bulk using ensemble plating assays. The results of these experiments considering the contribution of MscL to survival is shown in Figure 5 of their work, although with out displayed uncertainty in the survival probability. Figure S6B of their work shows the approximate error in survival probability through ensemble plating assays for three different strains (Fig. S15A), which is approximately 30%. Using this approximate error and the data shown in their Figure 5B, we have reproduced this plot with error bars in both measured dimensions (Fig. S15B). This plot shows that even when the mean survival probability is 100%, the variation in the measured survival probability is large, extending as low as ~70%. This variation is likely born from a multitude of experimental steps including time of outgrowth, variation in shock rate, plating efficiency, and counting errors. As our experimental approach directly measures the survival/death of individual cells, we remove many sources of error that would arise from an ensemble approach, albeit at lower throughput. While it is possible that the discrepancy between van den Berg et al. 2016 and the work under review could arise from other unknown factors, we believe that single-cell experiments introduce the fewest sources of error.

Supplement G: Strains

Strain name	Genotype	Reference
MJF641	Frag1, Δ*mscL::cm*, Δ*mscS*, Δ*mscK::kan*, Δ*ybdG::apr*, Δ*ynaI*, Δ*yjeP*, Δ*ybiO*, *ycjM::Tn10*	(16)
MLG910	MG1655, Δ*mscL* ::ϕ*mscL-sfGFP*, Δ*galK::kan*, Δ*lacI*, Δ*lacZYA*	(3)
D6LG-Tn10	Frag1, Δ*mscL* ::ϕ*mscL-sfGFP*, Δ*mscS*, Δ*mscK::kan*, Δ*ybdG::apr*, Δ*ynaI*, Δ*yjeP*, Δ*ybiO*, *ycjM::Tn10*	This work
D6LG (SD0)	Frag1, Δ*mscL* ::ϕ*mscL-sfGFP*, Δ*mscS*, Δ*mscK::kan*, Δ*ybdG::apr*, Δ*ynaI*, Δ*yjeP*, Δ*ybiO*	This work
XTL298	CC4231, *araD:: tetA-sacB-amp*	(17)
D6LTetSac	Frag1, *mscL-sfGFP:: tetA-sacB*, Δ*mscS*, Δ*mscK::kan*, Δ*ybdG::apr*, Δ*ynaI*, Δ*yjeP*, Δ*ybiO*	This work
D6LG (SD1)	Frag1, Δ*mscL* ::ϕ*mscL-sfGFP*, Δ*mscS*, Δ*mscK::kan*, Δ*ybdG::apr*, Δ*ynaI*, Δ*yjeP*, Δ*ybiO*	This work

Strain name	Genotype	Reference
D6LG (SD2)	Frag1, Δ*mscL ::ϕmscL-sfGFP,* Δ*mscS,* Δ*mscK::kan,* Δ*ybdG::apr,* Δ*ynaI,* Δ*yjeP,* Δ*ybiO*	This work
D6LG (SD4)	Frag1, Δ*mscL ::ϕmscL-sfGFP,* Δ*mscS,* Δ*mscK::kan,* Δ*ybdG::apr,* Δ*ynaI,* Δ*yjeP,* Δ*ybiO*	This work
D6LG (SD6)	Frag1, Δ*mscL ::ϕmscL-sfGFP,* Δ*mscS,* Δ*mscK::kan,* Δ*ybdG::apr,* Δ*ynaI,* Δ*yjeP,* Δ*ybiO*	This work
D6LG (12SD2)	Frag1, Δ*mscL ::ϕmscL-sfGFP,* Δ*mscS,* Δ*mscK::kan,* Δ*ybdG::apr,* Δ*ynaI,* Δ*yjeP,* Δ*ybiO*	This work
D6LG (16SD0)	Frag1, Δ*mscL ::ϕmscL-sfGFP,* Δ*mscS,* Δ*mscK::kan,* Δ*ybdG::apr,* Δ*ynaI,* Δ*yjeP,* Δ*ybiO*	This work

:*Escherichia coli* strains used in this work.

TABLE S4 Oligonucleotide sequences used in this work. Bold and italics correspond to Shine-Dalgarno sequence modifications and `AT` hairpin insertion modifications, respectively. Double bar `||` indicates a transposon insertion site.

<table>
<tr><th>Primer Name</th><th>Sequence (5′ → 3′)</th></tr>
<tr><td>Tn10delR</td><td>taaagccaacggcatccaggcggacatactcagca||
cctttcgcaaggtaacagagtaaaacatccaccat</td></tr>
<tr><td>MscLSPSac</td><td>gaaaatggcttaacatttgttagacttatggttgtcgg
cttcatagggagTCCTAATTTTTGTTGACACTCTATC</td></tr>
<tr><td>MscLSPSacR</td><td>accacgttcccgcgcatcgcaaattcgcgaaat
tctttaataatgctcatATCAAAGGGAAAACTGTCCATA</td></tr>
<tr><td>MscL-SD1R</td><td>atcgcaaattcgcgaaattctttaataatgctcat
gttattctcctcatgaagccgacaaccataagtctaacaaa</td></tr>
<tr><td>MscL-SD2R</td><td>atcgcaaattcgcgaaattctttaataatgctcatgttatt
tcccctatgaagccgacaaccataagtctaacaaa</td></tr>
<tr><td>MscL-SD4R</td><td>atcgcaaattcgcgaaattctttaataatgctcat
gttatt cctgctatgaagccgacaaccataagtctaacaaa</td></tr>
<tr><td>MscL-SD6R</td><td>atcgcaaattcgcgaaattctttaataatgctcat
gttatt gctcgtatgaagccgacaaccataagtctaacaaa</td></tr>
<tr><td>MscL-12SD2R</td><td>atcgcaaattcgcgaaattctttaataatgctcat
atatatatatat tcccctatgaagccgacaaccataagtctaacaaa</td></tr>
</table>

Primer Name	Sequence (5′ → 3′)
MscL-16SD0R	atcgcaaattcgcgaaattctttaataatgctcat
	atatatatatatatat
	**ctccct**atgaagccgacaaccataagtctaacaaa

References

1. Blount P, Sukharev SI, Moe PC, Martinac B, Kung C. 1999. Mechanosensitive channels of bacteria. Methods in Enzymology 294:458–482.

2. Blount P, Sukharev SI, Schroeder MJ, Nagle SK, Kung C. 1996. Single residue substitutions that change the gating properties of a mechanosensitive channel in Escherichia coli. Proc Natl Acad Sci U S A 93:11652–7.

3. Bialecka-Fornal M, Lee HJ, DeBerg HA, Gandhi CS, Phillips R. 2012. Single-Cell Census of Mechanosensitive Channels in Living Bacteria. PLoS ONE 7:e33077.

4. Milo R, Jorgensen P, Moran U, Weber G, Springer M. 2010. BioNumbersthe database of key numbers in molecular and cell biology. Nucleic Acids Research 38:D750–D753.

5. van den Berg J, Galbiati H, Rasmussen A, Miller S, Poolman B. 2016. On the mobility, membrane location and functionality of mechanosensitive channels in Escherichia coli. Scientific Reports 6.

6. Grage SL, Keleshian AM, Turdzeladze T, Battle AR, Tay WC, May RP, Holt SA, Contera SA, Haertlein M, Moulin M, Pal P, Rohde PR, Forsyth VT, Watts A, Huang KC, Ulrich AS, Martinac B. 2011. Bilayer-mediated clustering and functional interaction of MscL channels. Biophys J 100:1252–60.

7. Sivia D, Skilling J. 2006. Data Analysis: A Bayesian TutorialSecond Edition. Oxford University Press, Oxford, New York.

8. Anderson RP, Jin R, Grunkemeier GL. 2003. Understanding logistic regression analysis in clinical reports: An introduction. The Annals of Thoracic Surgery 75:753–757.

9. Mishra V, Skotak M, Schuetz H, Heller A, Haorah J, Chandra N. 2016. Primary blast causes mild, moderate, severe and lethal TBI with increasing blast overpressures: Experimental rat injury model. Scientific Reports 6:26992.

10. Stahler GJ, Mennis J, Belenko S, Welsh WN, Hiller ML, Zajac G. 2013. Predicting Recidivism For Released State Prison Offenders. Criminal justice and behavior 40:690–711.

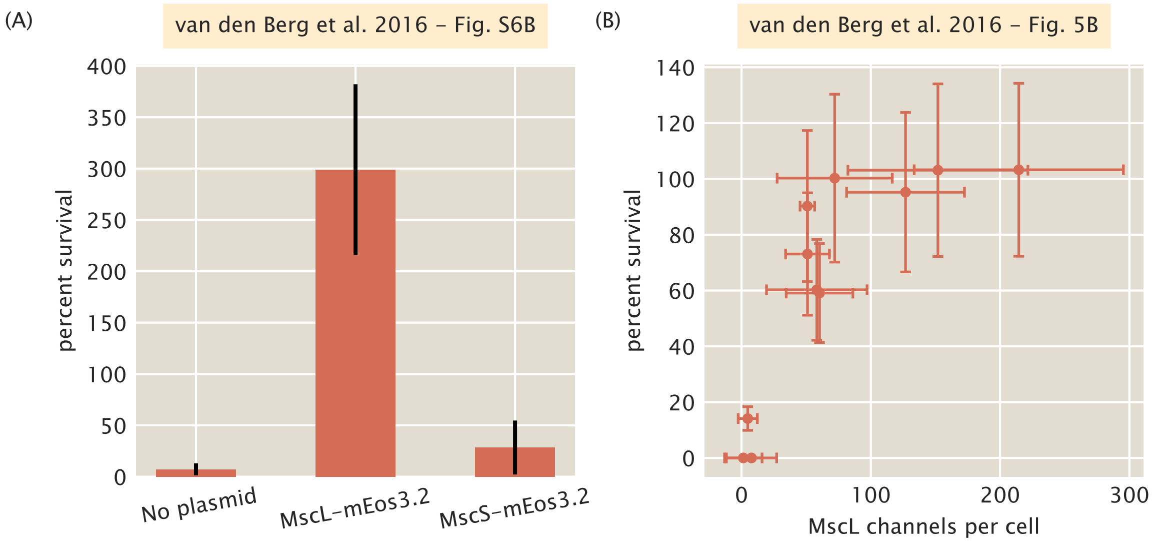

FIG S15 MscL abundance vs survival data reported in van den Berg et al. 2016 with included error. (A) Reported survival probabilities of a strain lacking all mechanosensitive channels (“no plasmid”), plasmid borne MscL-mEos3.2, and plasmid borne MscS-mEos3.2. Approximate reported errors for MscL-mEos3.2 survival probability is 30%. (B) The measurement of survival probability as a function of MscL channel copy number was obtained from Figure 5B in van den Berg et al 2016. Errors in channel copy number represent the standard deviation of several biological replicates (present in original figure) while the error in survival probability is taken as ~ 30%.

11. Cheng W, Hüllermeier E. 2009. Combining instance-based learning and logistic regression for multilabel classification. Machine Learning 76:211–225.

12. Dreiseitl S, Ohno-Machado L. 2002. Logistic regression and artificial neural network classification models: A methodology review. Journal of Biomedical Informatics 35:352–359.

13. Ng AY, Jordan MI. On Discriminative vs. Generative Classifiers: A comparison of logistic regression and naive Bayes 8.

14. Downey A. 2014. Probably Overthinking It: Bayes's theorem and logistic regression. Probably Overthinking It.

15. Bialecka-Fornal M, Lee HJ, Phillips R. 2015. The Rate of Osmotic Downshock Determines the Survival Probability of Bacterial Mechanosensitive Channel Mutants. Journal of Bacteriology 197:231–237.

16. Edwards MD, Black S, Rasmussen T, Rasmussen A, Stokes NR, Stephen TL, Miller S, Booth IR. Characterization of three novel mechanosensitive channel activities in Escherichia coli. Channels (Austin) 6:272–81.

17. Li X-t, Thomason LC, Sawitzke JA, Costantino N, Court DL. 2013. Positive and negative selection using the tetA-sacB cassette: Recombineering and P1 transduction in *Escherichia coli*. Nucleic acids research 41:e204–e204.
